# Elk1 affects katanin and spastin proteins via differential transcriptional and post-transcriptional regulations

**DOI:** 10.1371/journal.pone.0212518

**Published:** 2019-02-21

**Authors:** Dolunay Kelle, Koray Kırımtay, Ece Selçuk, Arzu Karabay

**Affiliations:** Department of Molecular Biology and Genetics, Istanbul Technical University, Maslak, Istanbul, Turkey; Virginia Commonwealth University, UNITED STATES

## Abstract

Microtubule severing, which is highly critical for the survival of both mitotic and post-mitotic cells, has to be precisely adjusted by regulating the expression levels of severing proteins, katanin and spastin. Even though severing mechanism is relatively well-studied, there are limited studies for the transcriptional regulation of microtubule severing proteins. In this study, we identified the main regulatory region of *KATNA1* gene encoding katanin-p60 as 5’ UTR, which has a key role for its expression, and showed Elk1 binding to *KATNA1*. Furthermore, we identified that Elk1 decreased katanin-p60 and spastin protein expressions, while mRNA levels were increased upon Elk1 overexpression. In addition, SUMOylation is a known post-translational modification regulating Elk1 activity. A previous study suggested that K230, K249, K254 amino acids in the R domain are the main SUMOylation sites; however, we identified that these amino acids are neither essential nor substantial for Elk1 SUMOylation. Also, we determined that *KATNA1* methylation results in the reduction of Elk1 binding whereas *SPG4* methylation does not. Together, our findings emphasizing the impacts of both transcriptional and post-transcriptional regulations of katanin-p60 and spastin suggest that Elk1 has a key role for differential expression patterns of microtubule severing proteins, thereby regulating cellular functions through alterations of microtubule organization.

## Introduction

Microtubules are responsible for many important cellular events in mitotic cells, such as cell division, localization of organelles and migration, as well as neuronal branching, axonal growth and neuronal morphogenesis in neurons. Assembly and disassembly property of microtubules, which is called “dynamic instability” is required to carry out these processes. In addition, microtubule severing proteins such as katanin and spastin promote the dynamic nature of microtubules by generating internal breakages within the microtubule lattice [1–4].

Katanin is a heterodimeric protein consisting of 60 kDa (katanin-p60) and 80 kDa (katanin-p80) polypeptides, which are encoded by *KATNA1* and *KATNB1* genes, respectively [2]. Spastin is a monomer encoded by *SPG4* (also known as *SPAST*) gene [5]. Both katanin and spastin proteins belong to ATPases associated with diverse cellular Activities (AAA) protein family [6].

Katanin-p60 has an AAA domain at the C-terminal to mediate enzymatic activities for severing of microtubules, while katanin-p80 contains WD40 repeat domain and procon80 domain [2]. Procon80 domain interacts with N-terminal of katanin-p60 and enhances its enzymatic activity. This domain also promotes the binding of katanin-p60 to microtubules by the inclusion of microtubule binding motif. WD40 domain is required for spindle pole or centrosome targeting and it acts as a negative regulator of microtubule severing by katanin-p60 [3,7]. The opposite effects of katanin-p80 domains determine the degree of which microtubules are severed since excessive severing is deleterious due to depletion of extreme microtubule mass [7–9].

Spastin has two domains at its C-terminal, which are AAA domain required for microtubule severing activity and Microtubule binding domain (MTBD), and there are Hydrophobic domain (HD) and Microtubule interacting and trafficking domain (MIT) domains at the N-terminal [10]. Spastin has two main isoforms as a result of two different initiation codons, which are M87 isoform (60 kDa) and M1 isoform (68 kDa). 86 residues at the N-terminal which corresponds to the HD are missing in the M87 isoform. Also, there are two additional 64 kDa and 55 kDa isoforms due to alternative splicing of *SPG4* exon 4 [11].

Microtubule severing is important for both mitotic and post-mitotic cells, as long microtubules are more stable than short ones and dynamic cellular processes require short microtubules. Microtubule severing is extremely critical in neurons since restraining of katanin and spastin activities leads to defects in axonal growth and neuronal processing [8,12]. Also, katanin-p60 mutations have been demonstrated to give rise to aberrant neuronal migration [13]. Even though both katanin and spastin are crucial for these activities, their severing mechanisms are partially different from each other. As a result of microtubule associated protein (MAP) protection, katanin leads to mixtures of both long and short microtubules specialized for axonal growth, whereas spastin severs microtubules into smaller pieces for branch formation regardless of MAPs [14,15]. Moreover, another mechanism underlying this difference might be the polyglutamylation of tubulins at the C-terminal. It has been shown that the long glutamate side chain induces spastin-mediated severing, whereas it has a weaker effect on katanin-p60 [16].

E Twenty Six-specific (ETS) domain proteins are essential for different processes in both adult and embryonic development. They act as nuclear targets of signal transduction pathways, such as p38, Jun N-terminal Kinase and Extracellular signal-regulated kinase (ERK) Mitogen-activated protein kinases (MAPKs) [17]. Ternary complex factors, a subgroup of ETS domain family, differ from other subgroups in terms of the presence of ETS domain at the N-terminal [18,19]. Elk1 is a member of this group and consists of five domains; A, B, C, D, and R. N-terminal A domain is the ETS DNA binding domain and has a repressor activity. B domain is responsible for the interaction with Serum Response Factor which allows ternary complex formation [20,21]. D domain is the docking site for MAPKs [22]. C domain at the C-terminal is the activation domain due to the presence of phosphorylation sites which are phosphorylated by MAPKs [23,24]. Although its activity is weaker than the A domain, R domain also plays a role in transcriptional repression through SUMOylation [25,26]. In mammalian cells, four small ubiquitin-like modifier (SUMO) isoforms exist; SUMO-1, SUMO-2, SUMO-3 and SUMO-4. SUMO-1 is ~45% identical to SUMO-2 and SUMO-3 which are ~95% identical with each other [27,28,29]. SUMO-4 is the subsequently identified isoform and it differs from the other isoforms in terms of its expression which is detected only in renal, immune, and pancreatic cells and also in human placenta [30].

Elk1 is mainly expressed in human brain and an important transcription factor for neurons [30,31]. Elk1 overexpression has been suggested to have impacts on neuronal viability in a localization-specific manner, presumably as a result of post-translational modifications [32,33]. In addition to viability, Elk1 has a role in neuronal differentiation as a transcription factor, causing an alteration in an immediate early gene (IEG) expression (*pip92*) through binding to its promoter region to induce pip92 expression [31]. Besides, co-localization of Elk1 and neuronal microtubules have already been shown in hippocampal and SH-SY5Y cells [34]. Due to playing important roles in neuronal differentiation and survival, also altering the transcriptional regulation of the genes related to neurodegenerative diseases, such as presenilin [35]; Elk1 might be a regulator of the expression of *KATNA1*, whose product katanin-p60 is highly critical for these neuronal processes which are directly affected in both differentiation and neurodegeneration. In addition, we have demonstrated the regulatory roles of Elk1 on other microtubule severing protein encoding genes; *SPG4* and *KATNB1* [36,37]. Many studies indicate that a transcription factor strongly influences target gene expression by binding to its conserved binding sites rather than non-conserved binding sites [38]. In our case, among the three theoretically identified Elk1 binding sites on *KATNA1*, one of them is highly conserved among species. Taken together, these findings indicate that Elk1 could have a role in *KATNA1* gene expression.

Here, we first identified the critical regions of *KATNA1* promoter for transcriptional regulation, then investigated the Elk1 binding to *KATNA1*. Furthermore, our previous study indicated the repressive effect of Elk1 on spastin expression, which was attributed to SUMOylation effect [36]. For this purpose, we also generated Elk1-3R construct including point mutations at the three lysine residues located at 230, 249, and 254 in the R domain, which have been previously reported as the main sites for SUMOylation [26]. We analyzed SUMOylation levels of wt-Elk1 and Elk1-3R and their effects on *KATNA1* and *SPG4* transcriptional regulations and protein expressions. Finally, we determined the effect of methylation at the Elk1 binding sites on *KATNA1* and *SPG4* for Elk1 binding.

## Materials and methods

### Materials

Dulbecco’s Modified Eagle’s Medium (DMEM) (4.5 g/L glucose w/L-glutamine) and Penicillin/Streptomycin were obtained from Lonza Ltd (Basel, Switzerland). Fetal Bovine Serum (FBS) was provided from Biowest (Nuaillé, France). ProtoScript II First Strand cDNA Synthesis Kit was purchased from New England Biolabs, Inc (Ipswich, MA, USA). NucleoSpin RNA Isolation Kit was supplied from Macherey-Nagel, GmbH & Co KG (Düren, Germany). LightCycler 480 Probes Master was obtained from Roche, Inc (Mannheim Germany). TransFast Transfection Reagent, Dual Luciferase Assay System and MagneHis Protein Purification System were purchased from Promega Corp. (Madison, USA). Polyethylenimine (PEI) (Linear, MW 25,000) was provided from Polysciences, Inc. (Warrington, PA). Elk1 and Elk1 mutated oligonucleotides were synthesized by Alpha DNA (Montreal, Quebec, Canada) and Macrogen, Inc (Amsterdam, The Netherlands). Biotin 3’ End DNA Labeling Kit, LightShift Chemiluminescent EMSA Kit and Chemiluminescent Nucleic Acid Detection Module Kit were purchased from Thermo Scientific Pierce, Inc (Rockford, USA). SimpleChIP Enzymatic Chromatin IP Kit (Magnetic Beads) was obtained from Cell Signaling Technology, Inc. iProof High-Fidelity PCR Kit was supported by Bio-Rad (California, USA). QuikChange II Site-Directed Mutagenesis Kit was purchased from Agilent Technologies, Inc (Santa Clara, CA, USA). NP-40 buffer was purchased from AppliChem (Darmstadt, Germany). Amaxa Cell Line Nucleofector Kit V and Nucleofector 2b was obtained from Lonza Ltd (Basel, Switzerland). Dynabeads Protein G was purchased from Novex Life Technologies supported by Invitrogen (Norway). ANTI-FLAG M2 Affinity Gel was supplied from Sigma-Aldrich, Inc (St. Louis, MO, USA).

### Bioinformatics

442 bp promoter region, 336 bp 5’ UTR region, 778 bp Promoter + 5’ UTR region and 3012 bp promoter + 5’ UTR + intron region of *KATNA1* gene were determined by using UCSC Genome Browser ‘Get Genomic Sequence Near Gene’ tool (http://genome.ucsc.edu/).

Both PROMO bioinformatics tool from ALGGEN server [39,40] (http://alggen.lsi.upc.es/cgi-bin/promo_v3/promo/promoinit.cgi?dirDB=TF_8.3) and MATCH public version 1.0 tool from geneXplain [41] (http://gene-regulation.com/cgi-bin/pub/programs/match/bin/match.cgi) were used to determine possible DNA-protein interaction sites within the promoter and 5’ UTR of *KATNA1* gene. Maximum matrix dissimilarity range was selected as 0–9%, and factor’s species and site species were chosen as *Homo sapiens* for PROMO. Also, “vertebrates” was selected as “group of matrices”, and “minimize false positives” option was selected as “cut-off selection for matrix group” for MATCH. Both *KATNA1* and *SPG4* sequences were analyzed for the presence of CpG island via EBI, EMBOSS CpGPlot/Report tool (http://www.ebi.ac.uk/Tools/seqstats/emboss_cpgplot/).

Theoretically determined possible Elk1 transcription factor binding sites were analyzed by UCSC Genome Browser (http://genome.ucsc.edu) Multiz Alignment of 100 Vertebrate, Basewise Conservation (phyloP) and Element Conservation (phastCons) for evolutionary conserved sequences among species.

### Plasmid constructs

*KATNA1* gene deletion constructs containing the regions described above were amplified by polymerase chain reaction (PCR). For this reaction, specific primers (Table 1) were synthesized by Alpha DNA and One Taq Enzyme System (NEB) was used as DNA polymerase and buffer solution. The amplified fragments were individually cloned into pGL3-basic luciferase reporter vector using SacI, HindIII, and XhoI cloning sites inside the vector. These constructs were used for luciferase experiments.

**Table 1:** Primer sequences for deletion constructs’ amplification.

Primer	Sequence
pGL3-KP_F	5’ AAAA**GAGCTC**GTGGAGATTGAGACTGGAGG 3’
pGL3-KP_R	5’ AAAA**AAGCTT**TCTTGCACCGCCTCCTCC 3’
pGL3-KU_F	5’ AAAA**AAGCTT**CTGCGGCGGCCCAAGCTC 3’
pGL3-KU_R	5’ AAAA**GAGCTC**ATGCGCACGCGCGGCCC 3’
pGL3-KPU_F	5’ AAAA**GAGCTC**GTGGAGATTGAGACTGGAGG 3’
pGL3-KPU_R	5’ AAAA**AAGCTT**CTGCGGCGGCCCAAGCTC 3’
pGL3-KPUI_F	5’ AAAA**GAGCTC**GTGGAGATTGAGACTGGAGG 3’
pGL3-KPUI_R	5’ AAAA**CTCGAG**CTAGGTGGGTTTTCTTTGCTTAG 3’

SacI, HindIII, and XhoI sites are shown in bold. Promoter, 5’ UTR and promoter + 5’ UTR sequences of *KATNA1* were clonned into pGL3 vector using SacI and HindIII, while promoter + 5’ UTR + intron sequence of *KATNA1* was cloned using SacI and XhoI restriction enzymes. KP:*KATNA1* Promoter, KU: *KATNA1* 5’ UTR, KPU: *KATNA1* Promoter + 5’ UTR, KPUI: *KATNA1* Promoter + 5’ UTR + Intron, F: Forward primer, R: Reverse primer.

The pCMV6_Elk1 construct (encoding amino acids 1–428), a gift from IAK described in our previous studies [34, 36, 37] was used in forced experiments, Western blotting, and Immunocytochemistry (ICC) experiments. The pRL-TK (Promega, E2241) was used as an internal control in luciferase assays.

### Cell culture

SH-SY5Y human neuroblastoma cells from ATCC CRL-2266 were cultured in high glucose DMEM supplemented with 10% FBS and penicillin/streptomycin at a final concentration of 100 μg/mL and cultivated at 37°C in an atmosphere of 5% CO_2_. Cells were seeded in 24-well plate at a density of 3×10^4^ cells/well for luciferase experiments, 5×10^5^ cells in 60 mm culture dish for Western blotting experiments, and 10^5^ cells in glass coverslips with 18 mm diameter.

### Transfection

For luciferase assays, TransFast Transfection Reagent was used at a ratio of 0.75 μg plasmid DNA: 4.6 μl TransFast reagent. Total plasmid DNA (Table 2) was mixed with appropriate amount of TransFast Transfection Reagent in DMEM and incubated for 15 min at room temperature. After removing the growth medium, transfection mixture was added to the cells and incubated for 1 h at 37°C, 5% CO_2_. Then, the transfection mixture was replaced with fresh growth medium and the cells were cultivated for 48 h.

**Table 2:** Plasmids used in transient transfection for luciferase assay.

Construct	Amount	Purpose
pGL3-test (KP,KU,KPU,KPUI)	800 ng	Luciferase assay
pGL3-basic	800 ng	Luciferase assay (internal control)
pRL-TK (*Renilla*)	30 ng	Luciferase assay (internal control)
pGL3-KPU	800 ng	Forced assay
pGL3-SP	800 ng	Forced assay
pCMV6_Elk1	300 ng	Forced assay
pCMV6_Elk1-3R	300 ng	Forced assay
pCMV6-Myc (empty)	300 ng	Forced assay

pGL3-test represents pGL3 vector containing four different constructs (KP:*KATNA1* Promoter, KU: *KATNA1* 5’ UTR, KPU: *KATNA1* Promoter + 5’ UTR, KPUI: *KATNA1)*. SP represents *SPG4* promoter.

For Western blotting experiments, cells were transfected with PEI at 1 μg plasmid DNA: 3 μl PEI ratio. Linear PEI was previously dissolved in 0.2N hydrochloric acid (HCl) at the final concentration of 1 mg/ml. 5 μg pCMV6_Elk1, 5 μg pCMV6_Elk1-3R, and 15 μl PEI (pH 1.5) (for each reaction) were separately mixed with 50 μl lactate buffer saline (LBS) including 20 mM sodium lactate (pH 4) and 150 mM sodium chloride, and incubated for 15 min at room temperature. DNA/LBS and PEI/LBS mixtures were combined and incubated for additional 15 min at room temperature. DMEM was mixed with transfection solution and growth medium of the cells was removed. Transfection solution was added to the cells and incubated for 1 h at 37°C, 5% CO_2_. Then, fresh growth medium was added and the cells were cultivated for 48 h.

For quantitative reverse transcription PCR (qRT-PCR) and immunoprecipitation (IP) experiments, Amaxa Cell Line Nucleofector Kit V was used according to the manufacturer’s instructions. 2×10^6^ SH-SY5Y cells were nucleofected with 4 μg either pCMV6_Elk1 or pCMV6_Elk1-3R constructs using G-004 program of Nucleofector 2b device (Lonza).

### RNA isolation and cDNA synthesis

21h after the nucleofection, total RNA was isolated using NucleoSpin RNA isolation kit, and Protoscript II First Strand cDNA Synthesis Kit was used for cDNA synthesis according to the manufacturers’ instructions.

### qRT-PCR

Probe 6 (cat.no. 04685032001) targeting *KATNA1* mRNA, Probe 7 (cat.no. 04685059001) targeting *SPG4* mRNA, and Probe 60 (cat.no. 04688589001) targeting *CDKN1B* mRNA were selected from Universal Probe Library Assay Design Center and purchased from Roche. Specific primers were synthesized by Alpha DNA and listed in Table 3. *ACTB* (ß-actin) and *GAPDH* were used as reference genes, and probes and primers were obtained from Roche. Light Cycler 480 Probes Master was used for the reaction according to the manufacturers’ instructions. Each reaction was added to each well of Light Cycler 480 Multiwell Plate (Roche) and loaded to Light Cycler 480 Instrument (Roche) by choosing Dual Color Hydrolysis Probe/UPL Probe. HEX (533–580) was used for reference genes, FAM (465–510) was used for targets. The values of test groups were calculated according to ΔΔCt method [42]. The method uses the equation 2^- ΔΔCT^ = [(C_T gene of interest_—C_T internal control_) _treated sample_—(C_T gene of interest_—C_T internal control_) _control sample_].

**Table 3:** Primer sequences for qRT-PCR.

Primer	Sequence
KATNA1_F	5’ GCGGACATTACCAACGTGT 3’
KATNA1_R	5’ CATGTGCATTTCTTCTTTGGAA 3’
SPG4_F	5’ AACCTTCTTTAATATAAGTGCTGCAAG 3’
SPG4_R	5’ AAGAGCCCTCACCAATTTCTC 3’
CDKN1B_F	5’ TTTGACTTGCATGAAGAGAAGC 3’
CDKN1B_R	5’ AGCTGTCTCTGAAAGGGACATT 3’

F: Forward primer, R: Reverse primer.

### Luciferase assays

Dual-Luciferase Reporter Assay System and Fluoroskan Ascent FL Luminometre (Thermo Electron Co., Hudson, USA) were used to measure renilla and firefly luciferase activities. At 48 h post transfection, growth medium was removed. 60 μl 1X passive lysis buffer was added to each well and incubated on a rocking platform shaker for 15 min. Cell lysates were combined sequentially with firefly and renilla specific substrates into luminometer plates according to the manufacturer’s instructions. Luminometer was programmed to perform a 2 sec pre-measurement delay while shaking the plate, followed by a 10 sec measurement period for each reporter assay. All experiments were performed in triplicates and were repeated 3 times using different DNA preparations.

### Site directed mutagenesis

Site directed mutagenesis was performed in order to generate pCMV6_Elk1-3R construct, which was previously identified as SUMO mutant [26]. Three amino acids at the R domain of Elk1 were converted from lysine to arginine (K230R, K249R, K254R). QuikChange II Site-Directed Mutagenesis Kit was used according to the manufacturer’s instructions. Primer sequences are shown at Table 4.

**Table 4:** Primer sequences for site directed mutagenesis.

Primer	Sequence
K230R_F	5’ CGAGGCCCCAAACCTGAGATCGGAAGAGCTTAATG 3’
K230R_R	5’ CATTAAGCTCTTCCGATCTCAGGTTTGGGGCCTCG 3’
K249R_F	5’ CTTTGCCCCCAGAAGTGAGAGTAGAAGGGCCC 3’
K249R_R	5’ GGGCCCTTCTACTCTCACTTCTGGGGGCAAAG 3’
K254R_F	5’TGAAAGTAGAAGGGCCCAGGGAAGAGTTGGAAGT 3’
K254R_R	5’ ACTTCCAACTCTTCCCTGGGCCCTTCTACTTTCA 3’

F: Forward primer, R: Reverse primer.

### Recombinant protein production

Recombinant Elk1 protein including ETS DNA binding domain (amino acids 1–96) was produced as previously described [36]. Briefly, a 288 bp sequence of human Elk1-db was amplified and expressed as 6XHis tagged fusion protein Elk1-db. Fusion protein was purified using Ni-NTA agarose method according to the manufacturers’ instruction (QIAGEN Inc., Valencia, CA, USA). Finally, purified Elk1-db was dialyzed using dialysis tubing cellulose membrane (Sigma-Aldrich Corp., St. Louis, MO, USA), and used in further electrophoretic mobility shift assay (EMSA) analysis.

### Biotinylation of oligonucleotides

Sequences of WT and mutant (Mut) oligonucleotides of *KATNA1* are shown in Table 5. Oligonucleotide probes were labeled separately using Biotin 3’ End DNA Labeling Kit that uses terminal deoxynucleotidyl transferase (TdT) to incorporate 1–3 biotinylated ribonucleotides onto the 3’ end of DNA strands. The reaction mixture was prepared according to the manufacturer’s instructions. For the purification of oligonucleotides, chloroform:isoamyl alcohol was used at 24:1 ratio, respectively. After the purification, separately labeled forward and reverse oligonucleotide probes were annealed at a ratio of 1:1 by heating to 95°C for 5 min, slow cooling by 2°C/min to their annealing temperature, annealing for 30 min and cooling to 4°C by 2°C/min.

**Table 5:** *KATNA1* oligonucleotides used in EMSA.

Oligo name	Sequence
Promoter_WT	5’ AGGGTGGAGATTGAGACTGGAGGAAGCCCTGTGCAGTACATAT 3’
UTR1_WT	5’ CGAGGTCGTCCCCGGCACCGGAAGTGACCCTGGCGGGTTTGT 3’
UTR2_WT	5’ GACTGGGTCAGGCCCTCCTTCCTCGCTGCCGGGATCTCCACTC 3’
Promoter_Mut	5' AGGGTGGAGATTGAGACCAAGAAGGACCCTGTGCAGTACATAT 3'
UTR1_Mut	5' CGAGGTCGTCCCCGGTGTTAAGGATGACCCTGGCGGGTTTGT 3'
UTR2_Mut	5' GACTGGGTCAGGCCCTCTCCTTCTATTGCCGGGATCTCCACTC-3'

Elk1 binding sites are shown in bold. WT represents wild-type. Purine-purine and pyrimidine-pyrimidine converted oligonucleotides in Elk1 binding sites are named as Mut.

### Methylation of oligonucleotides

M.SssI CpG methyltransferase (4U/μl, NEB) was used for methylation of double stranded and labeled probes of *KATNA1* and *SPG4* (Table 6). 500 ng biotinylated probes were incubated with 2.5 μl *M*.*Sss*I CpG methyltransferase, 5 μl diluted S-adenosylmethionine (1600 μM), 5 μl 10X NEBuffer 2 in a 50 μl reaction volume for 1 h at 37°C, then for 20 min at 65°C. Oligonucleotides were purified using chloroform:isoamyl alcohol at 24:1 ratio, respectively.

**Table 6:** Oligonucleotides used for methylation.

Oligo name	Sequence
KATNA1_UTR1_WT	5’ CGAGGTCGTCCCCGCGGCACCGCGGAAGTGACCCTGGCGCGGGTTTGT 3’
SPG4_WT_1	5’ TACGCGAAGGCTTCCTGGCAGGAGCTC 3’
SPG4_WT_2	5’ CGCGGAGAGGACAGCGCGACAGGAAGGGAGG 3’

Elk1 binding sites are shown in bold. CpG dinucleotides are underlined.

### Electrophoretic mobility shift assay

LightShift Chemiluminescent EMSA Kit was used for binding reactions. 20 fmol labeled oligonucleotides and 20 pmol unlabeled competitor oligonucleotides, as well as 20 fmol labeled and methylated or unmethylated oligonucleotides, were incubated with 300 ng Elk1-db protein in 1X binding buffer (pH 7.5, 10 mM Tris, 50 mM Potassium chloride (KCl)), 1 mM dithiothreitol, 1 μg Poly (dI•dC), 5% glycerol, 1 mM EDTA, 0.3% bovine serum albumin and 1X Protease Inhibitor Cocktail for 20 min at room temperature. Complexes and free probes were resolved on a 6% non-denaturating polyacrylamide gel in 0.5X Tris-Borate-EDTA by electrophoresis for 75 min at 90 V at 4°C. The separated bands on the gel were then transferred to Biodyne B Nylon Membrane (Pierce) using Trans-Blot SD (Bio-Rad) at 20V for 40 min at 4°C. Cross-link transfer of DNA to membrane was achieved by incubating the membrane with 254 nm UV bulbs for 12 min. In order to detect biotin-labeled DNA, Chemiluminescent Nucleic Acid Detection Module Kit was used and the membrane was visualized using C-DiGit Blot Scanner (LI-COR).

### Chromatin immunoprecipitation

Chromatin immunoprecipitation (ChIP) assay was performed using SimpleChIP Enzymatic Chromatin IP Kit (Magnetic Beads). To crosslink proteins to DNA, 37% formaldehyde was added to 4×10^7^ of SH-SY5Y cells at the final concentration of 1%. Chromatin digestion with 5 μl micrococcal nuclease was followed by sonication in order to break the nuclear membrane. Sonicator was programmed to perform 3 sets of 20 sec pulses at 30 W and 30 sec rest periods on ice. For IP reactions, each 10 μg digested chromatin was incubated with 10 μl positive control Histone H3, 1 μl negative control Normal Rabbit IgG, 2 μl Elk-1 antibody (Cell Signaling Technology) overnight at 4°C. 30 μl ChIP-Grade Protein G Magnetic Beads was added to each IP reactions and incubated for 2 h at 4°C. In order to remove non-associated DNA fragments, pellet protein G magnetic beads were washed. Protein/DNA complexes were eluted and DNA fragments were purified. iProof High-Fidelity PCR Kit was used for PCR amplification according to the manufacturer’s instructions. Specific primers for each Elk-1 binding sites were given at Table 7. PCR products were analyzed on agarose gel and the gel was visualized using ChemiDoc Imaging System (Bio-Rad).

**Table 7:** Primers used for PCR amplification of chromatin immunoprecipitation assay.

Primer	Sequence
Promoter_F	5’ GAAGTGCCCTATTCTGCCT 3’
Promoter_R	5’ TTCTCGGACACACTGCTAG 3’
UTR1_F	5’ CACACCCTCTTCCGCCGCT 3’
UTR1_R	5’ GCCCACTTTGCTCTCCGCTCA 3’
UTR2_F	5’ GTTGACGATTGAACTGGGCA 3’
UTR2_R	5’ TGAAAACTCACCTGCGGC 3’

F: Forward primer, R: Reverse primer.

### Whole cell extract

pCMV6_Elk1 or pCMV6_Elk1-3R transfected cells were extracted using NP-40 buffer containing 150 mM sodium chloride, 1% NP-40 and 50 mM Tris (pH 8.0).

### Western blotting

At 48 h post transfection of 5×10^5^ SH-SY5Y cells, total protein was extracted. Equal amounts (15 μg protein/lane) of proteins obtained from whole cell extracts of untransfected and Elk1 transfected samples were separated by SDS-PAGE and transferred onto nitrocellulose membrane by Trans-Blot Turbo Blotting System (Bio-Rad). Membrane was blocked in 5% skim milk powder/TBST (Tris buffered saline (TBS) containing 0.1% Tween 20) for 1 h at room temperature, and then incubated with the following primary antibodies at indicated dilutions overnight at 4°C; rabbit monoclonal His-tag antibody (1:1000, Cell Signaling Technology), mouse monoclonal spastin antibody (1:1000, Sigma), rabbit polyclonal katanin-p60 antibody (1:1000, ATLAS), mouse monoclonal p27 antibody (1:500, Santa Cruz), mouse monoclonal HuR antibody (1:500, Santa Cruz), mouse monoclonal β-Actin antibody (1:1000, Cell Signaling Technology), rabbit monoclonal GAPDH antibody (1:1000, Cell Signaling Technology), rabbit monoclonal β-tubulin antibody (1:1000, Cell Signaling Technology) in 5% skim milk powder/TBST. Membranes were then incubated with Horseradish peroxidase conjugated goat anti-rabbit or anti-mouse IgG secondary antibodies (1:3000, Cell Signaling Technology) for 1 h at room temperature. Bands were visualized using Visualizer Western Blot Detection Kit (Millipore) and ChemiDoc Imaging System (Bio-Rad).

### Immunocytochemistry

Nucleofected SH-SY5Y cells (10^5^ cells/well) were plated on poly-L-lysine coated coverslips. After 21 h, cells were fixed with 4% paraformaldehyde solution for 10 min at room temperature and treated with 0.1% saponin solution for 10 min at room temperature. Then, the cells were blocked with 3% Bovine serum albumin and 0.1% saponin solution for 1 h at room temperature. Rabbit polyclonal katanin-p60 antibody in 1:100 dilution (ATLAS) and mouse monoclonal FLAG-Tag antibody in 1:400 dilution (Cell Signaling Technology) were prepared in blocking solution and incubated with the cells overnight at +4°C. Following day, Alexa Fluor 647 anti-rabbit and Alexa Fluor 488 anti-mouse secondary antibodies (Cell Signaling Technology) were incubated with the cells in 1:200 dilutions for 1 h at room temperature in dark. ProLong Diamond Antifade Mountant with DAPI (Invitrogen Corp., Carlsbad, CA, USA) was used for mounting of coverslips and Leica TCS SP2 SE Confocal Microscope (Buffalo Grove, IL, USA) was used for the visualization. All images were taken using 63X objective at zoom 2.6.

### Immunoprecipitation

Before IP experiments, cells were transfected with PEI as described previously. 24 h post-transfection KCl treatment was performed. KCl solution (170 mM KCl, 2 mM CaCl_2_, 1 mM MgCl_2_, 10 mM HEPES) was added to fresh culture medium as the final volume of 31% in order to adjust the final concentration of KCl as 50 mM. 2 ml treatment mix was added to each 60 mm dish and the cells were incubated 1 h at 37°C, 5% CO_2_ culture incubator. After 1 h, the solution was discarded and the cells were incubated with fresh culture medium for 24 h at 37°C, 5% CO_2_ culture incubator. 48 h post-transfection, whole cell extract was isolated as described previously. To ensure expression after the transfection, SDS-PAGE and Western blotting were performed using rabbit monoclonal His-tag antibody (1:1000, Cell Signaling Technology), and rabbit monoclonal GAPDH antibody (1:1000, Cell Signaling Technology) was used as loading control.

For IP reactions with His-tag antibody, 700 μg of either wt-Elk1 or Elk1-3R transfected SH-SY5Y cell lysates were separately incubated with 3 μg rabbit monoclonal His-tag antibody (Cell Signaling Technology). For non-specific IgG and no antibody (beads only) reactions as the negative controls; the same amount of lysate was mixed with 3 μg normal rabbit IgG (supported by SimpleChIP Enzymatic Chromatin IP Kit) or the lysate was used without antibody, respectively. Mixtures were incubated overnight on a rotator at 6 rpm and 4°C. The next day, each reaction was combined with 50 μl Protein G Magnetic Beads and incubated for 1 h on a rotator at 7 rpm and room temperature. The beads were resuspended in 20 μl elution buffer (50 mM glycine, pH:2.8) and mixed with 2 μl 1 M Tris-HCl (pH:7.5).

IP using MagneHis Protein Purification System was performed with 700 μg wt-Elk1 and Elk1-3R overexpressed cell lysates, separately. Before the experiment, 1 ml MagneHis Binding/Wash Buffer was mixed with NaCl at a final concentration of 500 mM and 22 μl of 1 M imidazole (pH:8.0). MagneHis Binding/Wash Buffer was separately added to cell lysates to obtain 1.1 ml total volume. 60 μl MagneHis Ni-Particles was added and incubated for 5 h on a rotator at 8 rpm and 4°C. Then, nickel particles were washed 3 times using magnetic separation rack with MagneHis Binding/Wash Buffer, which was prepared as previously indicated. Finally, nickel particles were resuspended in 50 μl MagneHis Elution Buffer.

Another IP experiment was carried out with 700 μg wt-Elk1 and Elk1-3R overexpressed cell lysates by mixing 20 μl ANTI-FLAG M2 Affinity Gel. The reactions were incubated for 18 h at 4°C on a rotator at 8 rpm. The following day, FLAG-tag resin was washed 4 times with NP-40 by centrifuging at 2000 g for 2 min. It was resuspended in 50 μl 1.5X SDS sample buffer and boiled at 95°C for 10 min. Then, it was centrifuged at 5000 g for 2 min and the eluate was collected in a new tube. 5 μl of 1.5 M dithiothreitol was added to each sample and boiled at 95°C for 5 min.

IP samples and 2% input were separated by SDS-PAGE and Western blotting was performed as described previously. Rabbit monoclonal SUMO-1 antibody (1:500, Cell Signaling Technology), mouse monoclonal His-tag antibody (1:1000, Invitrogen), IRDye 800CW Goat anti-Rabbit IgG (H + L) (1:15000, LI-COR), and IRDye 680RD Goat anti-Mouse IgG (H + L) (1:15000, LI-COR) were used. The detection was performed using Odyssey CLx Imaging System (LI-COR).

### Integrative pixel analysis

Adobe Photoshop CC Software was used to analyze the relative intensities of the protein bands in Western blot images by measuring the selected band area and pixel values. These values were normalized to the internal control values. For Western blot images of IP experiments, each SUMOylation level was normalized to precipitation level of same reaction. Fiji extension of ImageJ software was used to measure integrated densities of ICC images. The measurement of integrated densities was followed by normalization of katanin-p60 densities to Elk1 densities in each cell in order to eliminate differential overexpression of Elk1.

### Statistical analysis

The experiments were statistically analyzed by two-tailed Student’s t-test using GraphPad Prism 6 software. Error bars in the graphs were generated using SEM values since the experiments were repeated in different days. Asterisk symbol (*) represents that the p-value is <0.05 meaning a statistically meaningful difference and NS represents non-significant differences.

## Results

### 5’ UTR is the major regulator sequence for the expression of *KATNA1* gene

3012 bp region of *KATNA1* gene (containing part of the promoter, 5’ UTR and intron) was determined via University of California Santa Cruz (UCSC) Bioinformatics Genome Browser software (http://genome.ucsc.edu/cgi-bin/hgGateway).

**Fig 1:**
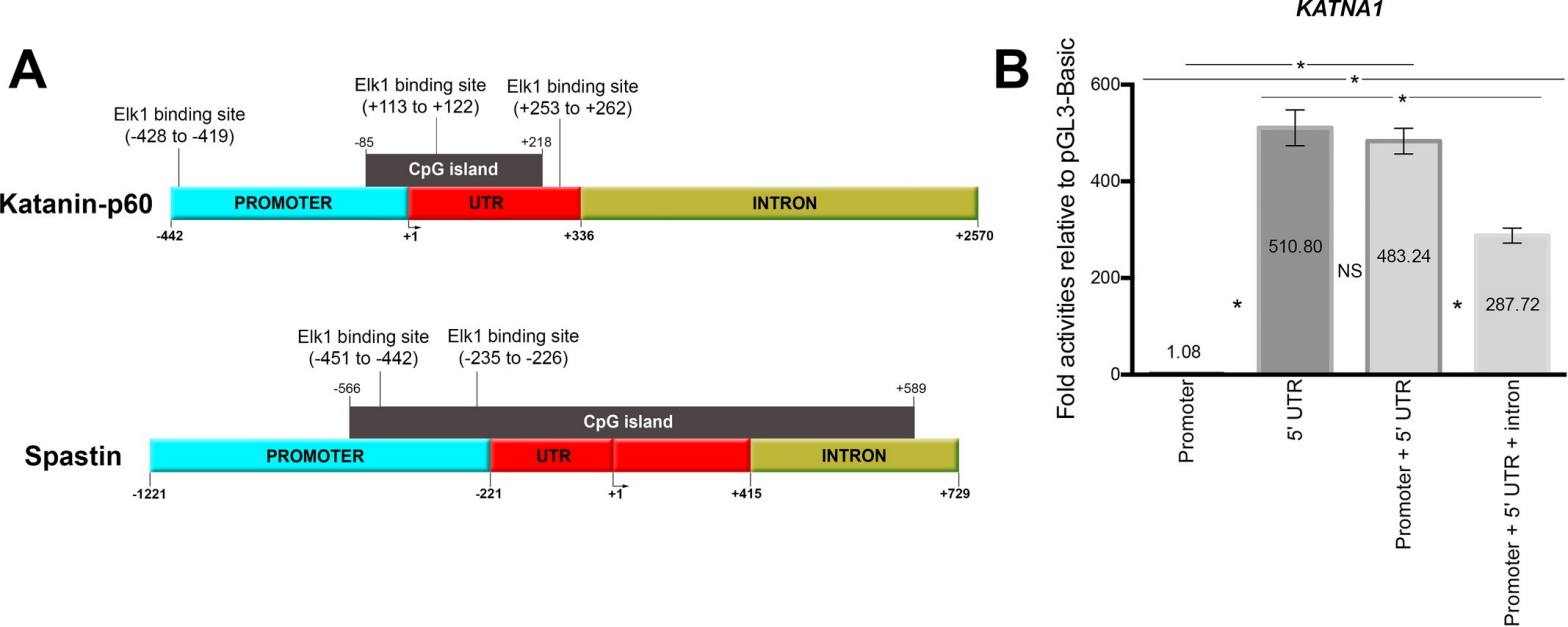
Schematic illustration of deletion constructs and their characteristics, and functional analysis of *KATNA1* regions. (A) Upper part of the figure represents the deletion constructs of *KATNA1* including promoter (442 bp), 5’ UTR (336 bp), promoter + 5’ UTR (778 bp), promoter + 5’ UTR + intron (3012 bp). Bioinformatic tools indicated three putative Elk1 binding sites on these regions; one of them is present on the promoter, other two sites are located on the 5 ‘UTR. Also, a CpG island is found between -85 and +218. Lower part of the figure shows *SPG4* promoter including two Elk1 binding sites which were previously identified [36]. A CpG island is located on the region from -566 to +589. (B) 5’ UTR has the highest activity and the difference between “promoter + 5’ UTR” and the “5’ UTR only” is not significant. SEM values are 0.05, 36.83, 26.29, 15.49, respectively. Each experiment was performed as triplicates on the same day and the experiments were repeated four times on separate days, independently (n = 4).

To define the critical region which has a key role in *KATNA1* gene expression, four deletion constructs were generated including promoter (442 bp), 5’ UTR (336 bp), promoter + 5’ UTR (778 bp), promoter + 5’ UTR + intron (3012 bp) sequences (Fig 1A).

All fragments were cloned into pGL3-basic vector one by one and the activity of deletion constructs was measured through the expression of luciferase gene following regulatory sequences indicated above. The measurements were normalized to the activity of empty pGL3-basic vector in order to determine fold activity.

The luciferase assay results (Fig 1B) indicated that the promoter had no effect on the expression of the downstream gene, however, the 5’ UTR construct showed the highest activity. Also, the difference between 5’ UTR and promoter + 5’ UTR constructs was not significant, indicating the ineffectiveness of the promoter on the downstream gene expression. The promoter + 5’ UTR + Intron construct had lower activity compared to the 5’ UTR and promoter + 5’ UTR constructs, concluding that 5’ UTR is the major regulator sequence for the expression of *KATNA1* gene. Since we aimed to determine the optimal regulatory region of *KATNA1*, the construct containing promoter + 5’ UTR sequence was used in further experiments. On the other hand, -1221 to -221 promoter region without 5’ UTR had been determined as the main regulator region of *SPG4* in our previous study (Fig 1A) [36].

### Elk1 binds to 5’ UTR of *KATNA1* gene

Since 5’ UTR is the critical site for *KATNA1* gene expression regulation, we theoretically identified Elk1 binding sites on the promoter and 5’ UTR of *KATNA1* using two bioinformatics tools. PROMO tool predicted three binding sites positioned at -428/-419, +113/+122, and +253/+262 (upper part of Fig 1A). However, MATCH tool indicated only one binding site positioned at +113/+122, which is the only shared predicted site with PROMO. All three predicted binding sites were analyzed for evolutionary conservation using UCSC Genome Browser (http://genome.ucsc.edu) Multiz Alignment of 100 Vertebrate, Basewise Conservation (phyloP) and Element Conservation (phastCons), and only one binding site positioned at +113/+122 was found to be conserved among species (S1 Fig).

**Fig 2:**
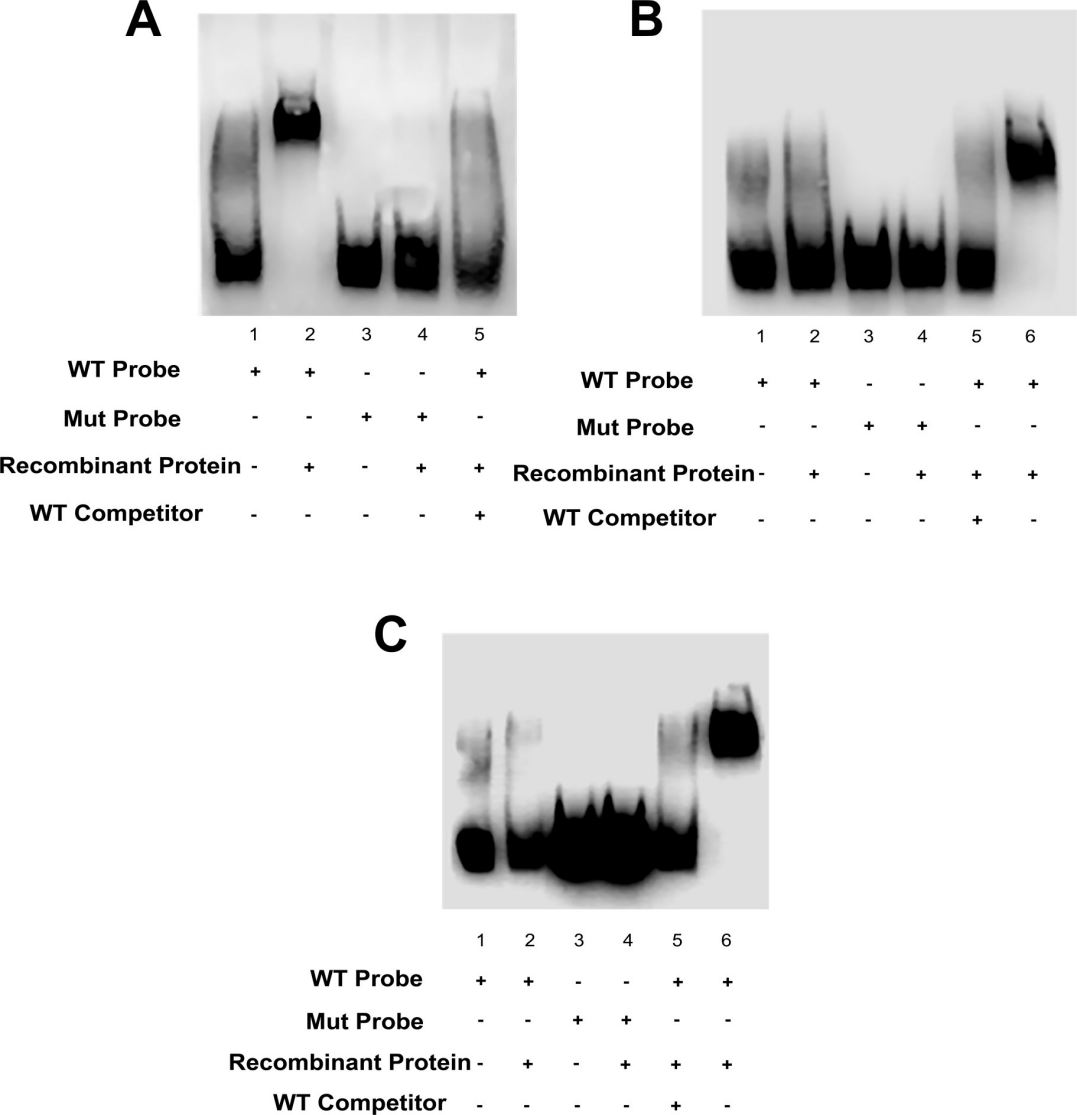
EMSA results. (A) Labeled WT and Mut oligos of the second binding site (+113/+122) on the 5’ UTR are shown in lane 1 and 3. In the presence of Elk1-db, clear complex formation of Elk1-db and WT probe is observed (lane 2). Also, there is no shift in the reaction in which Mut probe is incubated with Elk1-db (lane 4) and competition (lane 5), as expected. (B) Either WT or Mut oligonucleotides of the third putative binding site (+253/+262) could not participate in any complex formation with Elk1-db protein (lane 2 and 4, respectively). Lane 1 and 3 represent WT and Mut probes both of which lack Elk1-db, and positive control reaction is indicated in lane 6. (C) Biotin-labeled WT and Mut probes of the predicted promoter binding site (-428/-419) are loaded as controls (lane 1 and 3, respectively). When Elk1-db protein is incubated with both probes, no shift is observed with either WT probe (lane 2) or Mut probe (lane 3) and in competition reaction (lane 5) which included 1000-fold molar excess of unlabeled WT probe. Positive control reaction in which the complex formation is observed is indicated in lane 6.

After bioinformatic analyses, EMSA was performed in order to verify Elk1 binding to these theoretically predicted sites on *KATNA1*. Three putative binding sites positioning at -428/-419 (1), +113/+122 (2), +253/+262 (3) were used and binding ability of Elk1 to these sites is shown in Fig 2. Free wild-type and free mutated oligonucleotides used as controls are shown in lane 1 and 3, respectively in Fig 2A, 2B and 2C. In the presence of pure Elk-1-db protein and +113/+122 binding site including oligonucleotides, the shifted band indicating complex formation is clearly seen in lane 2 (Fig 2A). Additionally, no complex formation with mutated oligonucleotides in lane 4 or in competition reaction in lane 5 was observed (Fig 2A). These results indicated that Elk1 binds to +113/+122 site, which is located on 5’ UTR of *KATNA1*. Thus, this site was used as positive control for the remaining experiments performed for the other binding sites. Interestingly, Elk1-db protein could not bind to either +253/+262 site (Fig 2B) located on the 5’ UTR or -428/-419 site (Fig 2C) located on the promoter since no complex formation was observed with either wild-type probes in lane 2 in Fig 2B and 2C or mutated probes in lane 4 in Fig 2B and 2C. However, Elk1 binding was confirmed in positive controls (lanes 6 in Fig 2B and 2C).

**Fig 3:**
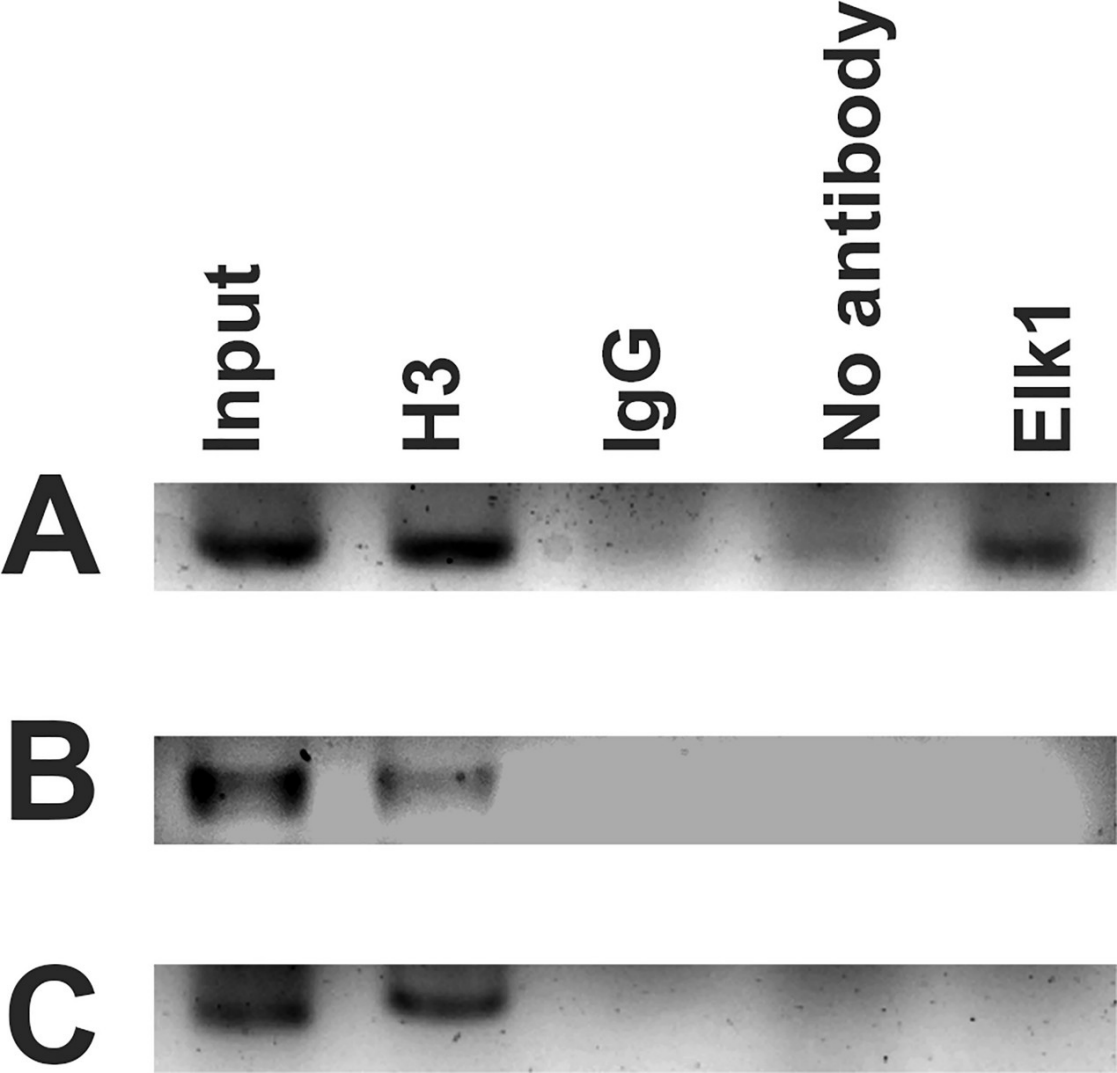
PCR results of ChIP assay. ChIP assay was performed with SH-SY5Y cells. 2% input DNA; H3 (positive control), rabbit IgG (negative control), no antibody (beads only, negative control), and Elk1 precipitated reactions were amplified with specific primers for each binding sites separately. ChIP results for +113/+122 binding site (A), +253/+262 binding site (B), and -428/-419 binding site (C) are indicated.

ChIP assay was also performed for these three binding sites in order to confirm Elk1 binding *in vivo*. The bands which indicate amplification of Elk1 binding sequence are seen in PCR results of input, positive control H3 and Elk1 precipitated reactions for +113/+122 binding site (Fig 3A). Also, the band was not observed in Elk1 precipitated reactions for +253/+262 (Fig 3B) and 428/-419 (Fig 3C) binding sites, while it was present in input and H3 reaction (Fig 3B and 3C). Therefore, ChIP results suggested that Elk1 could bind only to +113/+122 site of *KATNA1*. In addition, we have previously demonstrated Elk1 binding to *SPG4* promoter at two different sites located on -451/-442 and -235/-226 (Fig 1A) [36].

### Elk1 promotes mRNA levels of *KATNA1* and *SPG4*

**Fig 4:**
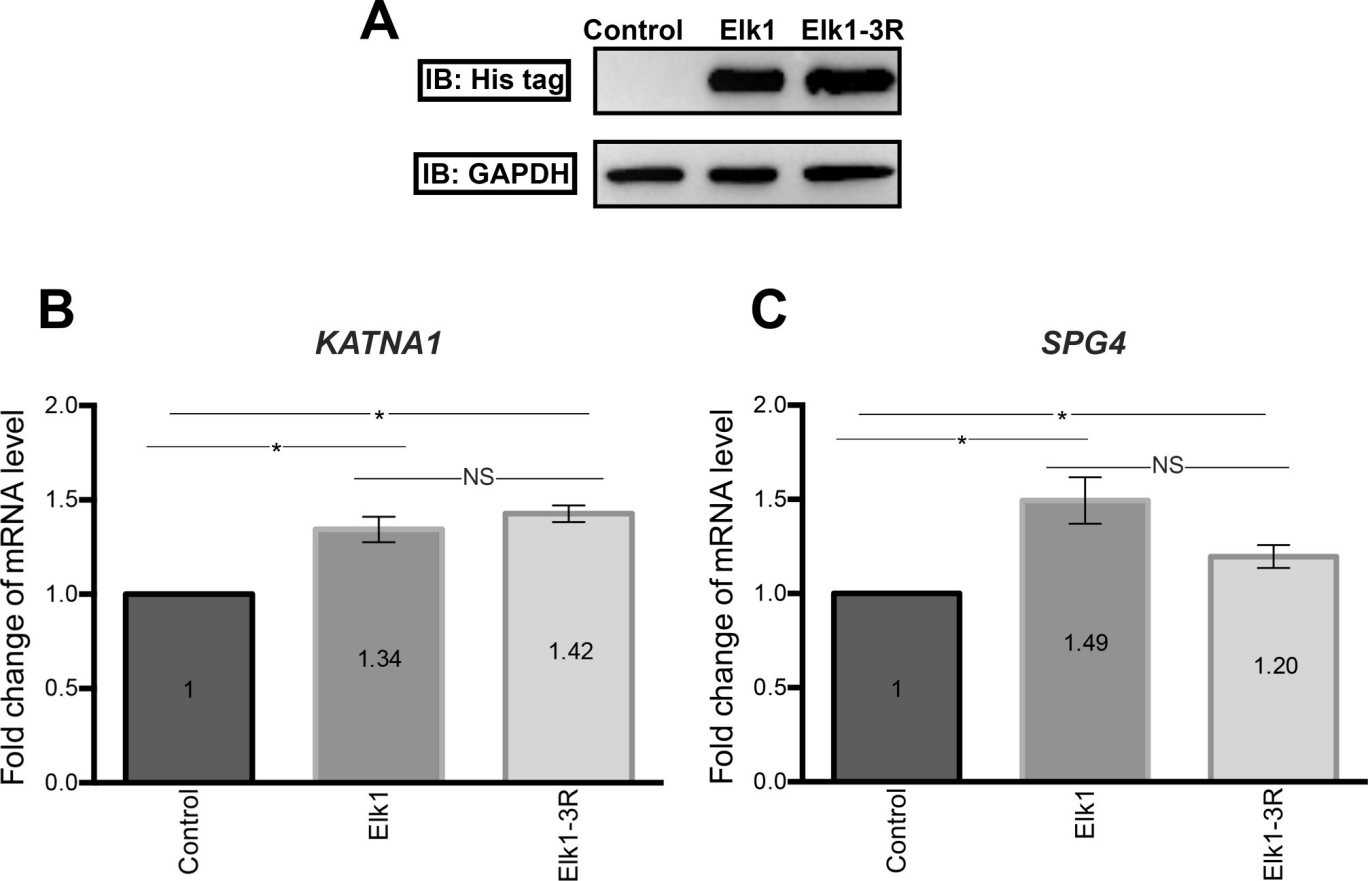
qRT-PCR results. (A) Transfection of SH-SY5Y cells with either Elk1 or Elk1-3R constructs was controlled for similar expression levels of both. (B) Both Elk1 and Elk1-3R promote the transcription of *KATNA1*, significantly. SEM values for control, Elk1, and Elk1-3R experiments are 0, 0.07, 0.04, respectively. Each experiment was performed as triplicates on the same day and the experiments were repeated three times on separate days, independently (n = 3). (C) mRNA level of *SPG4* is increased due to Elk1 and Elk1-3R. SEM values for control, Elk1, and Elk1-3R experiments are 0, 0.12, 0.06, respectively. Each experiment was performed as triplicates on the same day and the experiments were repeated three times on separate days, independently (n = 3).

After transfection of SH-SY5Y cells with wt-Elk1 and Elk1-3R constructs, total RNA was isolated and cDNA was synthesized. Elk1 overexpression in these cells were validated by Western blotting (Fig 4A). Then, mRNA levels of *KATNA1* and *SPG4* were measured by qRT-PCR. The results showed that both Elk1 and Elk1-3R increased the level of *KATNA1* (Fig 4B) and *SPG4* (Fig 4C) mRNAs. However, no significant difference was detected between the activities of Elk1 and Elk1-3R for the regulation of both *KATNA1* and *SPG4* transcripts.

### Elk1 represses katanin-p60 and spastin proteins

To ascertain the effect of Elk1 and Elk1-3R on *KATNA1* and *SPG4* regulatory regions, we performed forced experiment by luciferase reporter assay through co-transfection of SH-SY5Y cells with either pCMV6_Elk1 or pCMV6_Elk1-3R and pGL3-KPU or pGL3-SP constructs. Also, pGL3-basic, pGL3-KPU and pGL3-SP with pCMV-empty vector; pGL3-basic with pCMV6_Elk1 or pCMV6_Elk1-3R were co-transfected separately for normalization and calculation of the fold activity.

**Fig 5:**
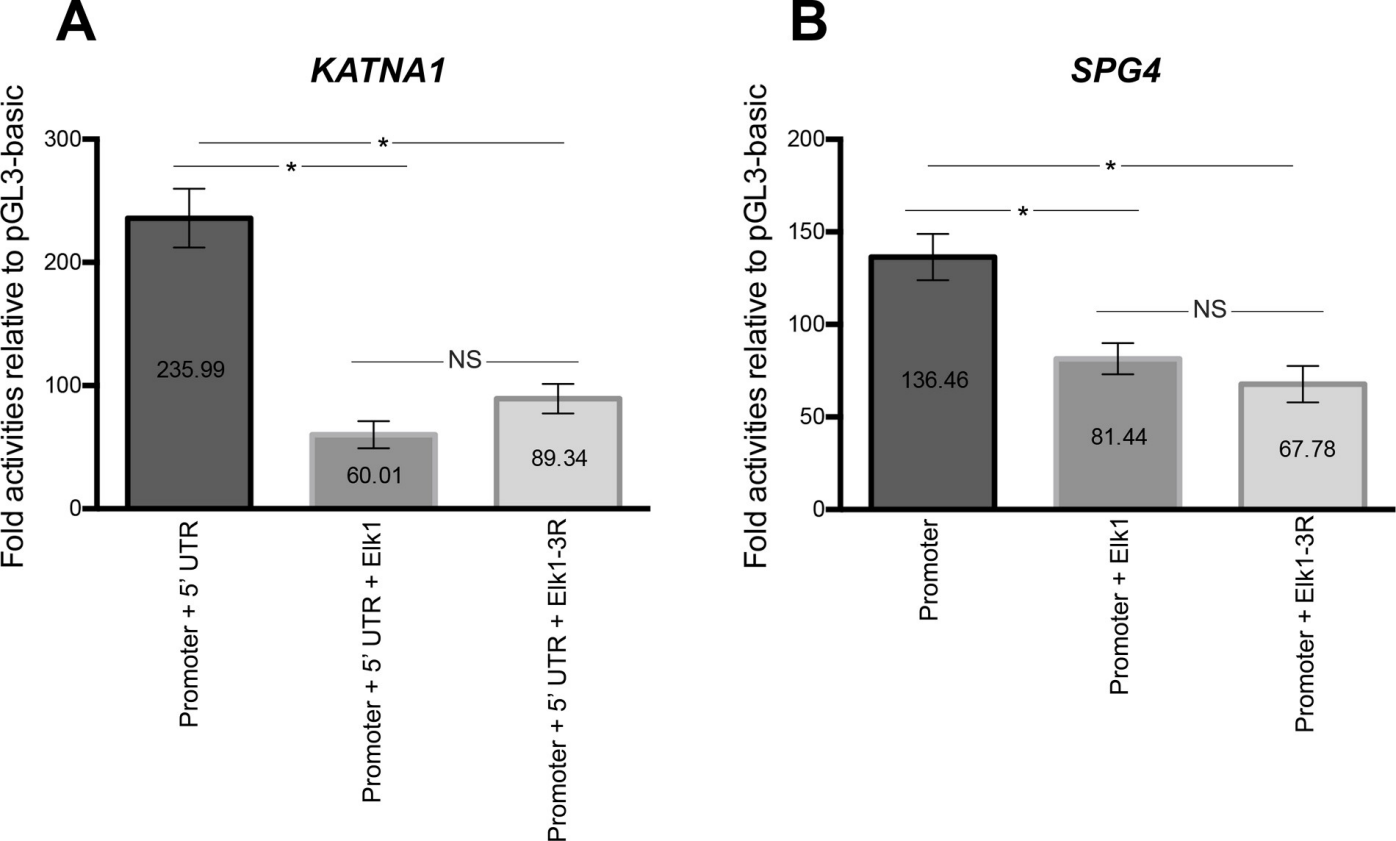
Luciferase assay results of *KATNA1* and *SPG4*. **(**A) In order to ascertain the effect of Elk1 on *KATNA1* regulatory regions, SH-SY5Y cells were co-transfected with pGL3-KPU construct and with either pCMV6_Elk1 or pCMV6_Elk1-3R. Also, pGL3-basic and pCMV6-empty vector co-transfection was performed. Luciferase assay was performed 48 h post-transfection, and calculated fold activities indicated that wt-Elk1 significantly decreased promoter + 5’ UTR activity of *KATNA1*. Even though Elk1-3R caused a partial increase compared to wt-Elk1, it was not statistically meaningful. Thus, it also significantly decreased promoter + 5’UTR activity compared to unforced (in the absence of Elk1) experiment. SEM values are 23.79, 10.99 and 11.92, respectively. Each experiment was performed as triplicates on the same day and the experiments were repeated three times on separate days, independently (n = 3). (B) For the same purpose, SH-SY5Y cells were transfected with pGL3-SP and with either pCMV6_Elk1 or pCMV6_Elk1-3R and pGL3-SP with pCMV6-empty vector. Both WT-Elk1 and Elk1-3R significantly decreased the promoter activity of *SPG4*. Even though Elk1-3R decreased the activity more than wt-Elk1, the difference was not significant. SEM values are 12.51, 8.41 and 9.83, respectively. Each experiment was performed as triplicates on the same day and the experiments were repeated three times on separate days, independently (n = 3).

As compared to controls, Elk1 significantly decreased reporter protein level of pGL3-KPU construct containing *KATNA1* promoter + 5’ UTR (Fig 5A). Even though Elk1-3R caused a slight increase in the expression, it was not significant in comparison to Elk1. Thus, there was no difference between the regulatory effects of Elk1 and Elk1-3R on *KATNA1* in a statistically meaningful manner. The reporter protein expression of pGL3-SP construct containing *SPG4* promoter was also repressed by Elk1. Even though Elk1-3R seemed to decrease the expression more than Elk1, this slight difference was not significant (Fig 5B).

**Fig 6:**
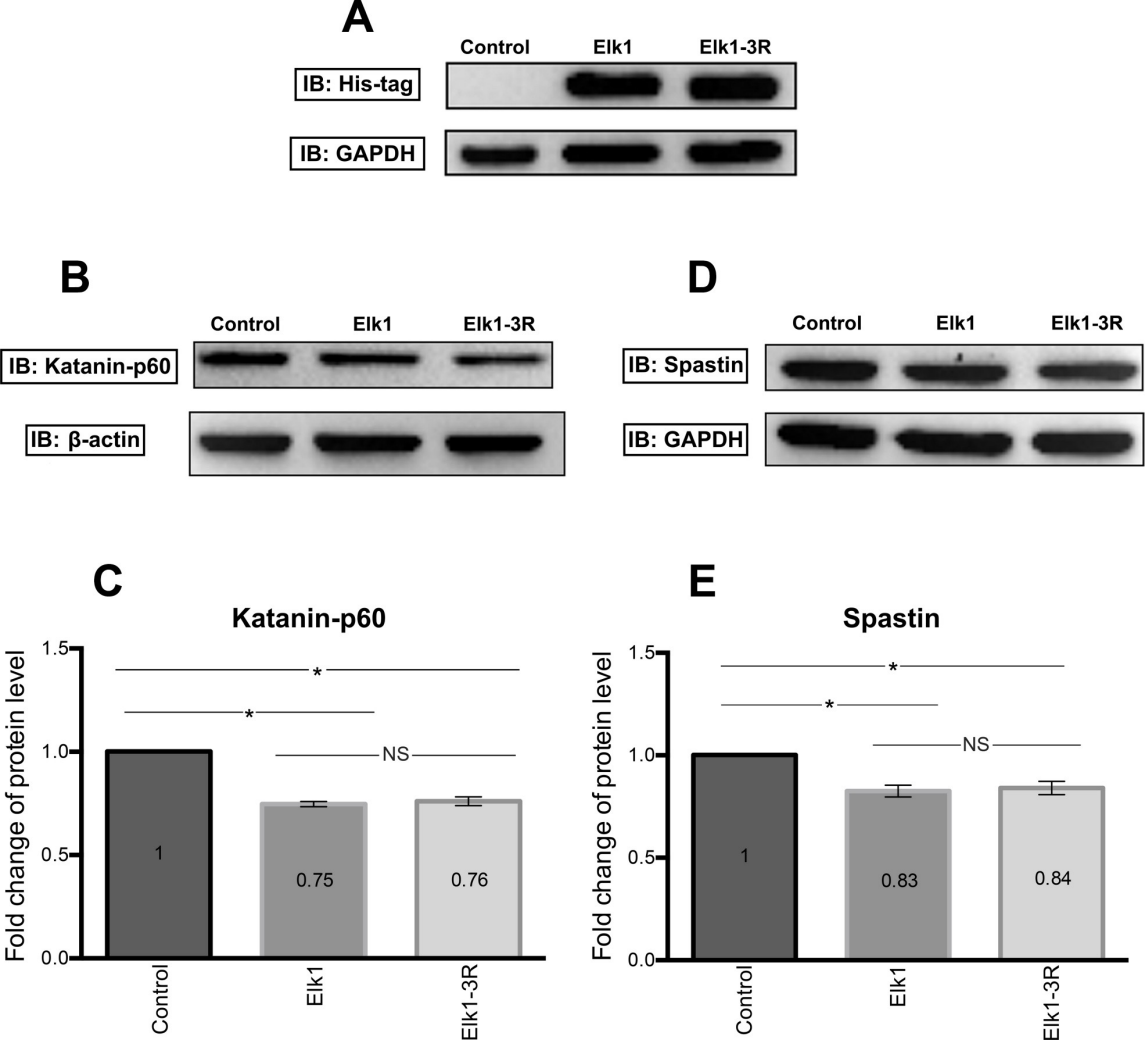
Western blotting results of Elk1 and Elk1-3R effects on both endogenous katanin-p60 and spastin proteins. (A) Analysis of SH-SY5Y transfection efficacy with His-tag including pCMV6_Elk1 and pCMV6_Elk1-3R vectors. (B) Western blotting results showing katanin-p60 level in untransfected (control), pCMV6_Elk1 transfected, and pCMV6_Elk1-3R transfected SH-SY5Y cells. Transfection experiment was performed two times on separate days and Western blotting experiment was performed two times with each sample. (C) Quantification of katanin-p60 level was performed by normalizing band intensities of katanin-p60 to band intensities of ß-actin. Results indicated that both Elk1 and Elk1-3R decreased the katanin-p60 level significantly and the difference between them was not significant. SEM values are 0, 0.01, and 0.02, respectively (n = 4). (D) Western blotting results showing spastin level in untransfected (control), pCMV6_Elk1 transfected, and pCMV6_Elk1-3R transfected SH-SY5Y cells. Transfection experiment was performed two times on separate days and Western blotting experiment was performed two times with each sample. (E) Quantification of spastin level was done as previously described. Results indicated that there was no significant spastin level difference between Elk1 and Elk1-3R transfected cells. However, they were significant compared to untransfected cells. SEM values are 0, 0.03, and 0.03, respectively (n = 4).

Forced experiment which indicated the regulatory region effects on reporter protein was followed by determination of the endogenous katanin-p60 and spastin protein levels upon Elk1 and Elk1-3R overexpression. To do this, endogenous katanin-p60 and spastin protein levels in untransfected (control), Elk1 transfected, and Elk1-3R transfected SH-SY5Y cells were analyzed by Western blotting. Expressions of the transfected constructs were confirmed by targeting His-tag of pCMV6 vectors (Fig 6A).

Results showed that both katanin-p60 (Fig 6B and 6C) and spastin (Fig 6D and 6E) protein levels were decreased in Elk1 and Elk1-3R overexpressed cells and there was no significant difference between Elk1 and Elk1-3R.

**Fig 7:**
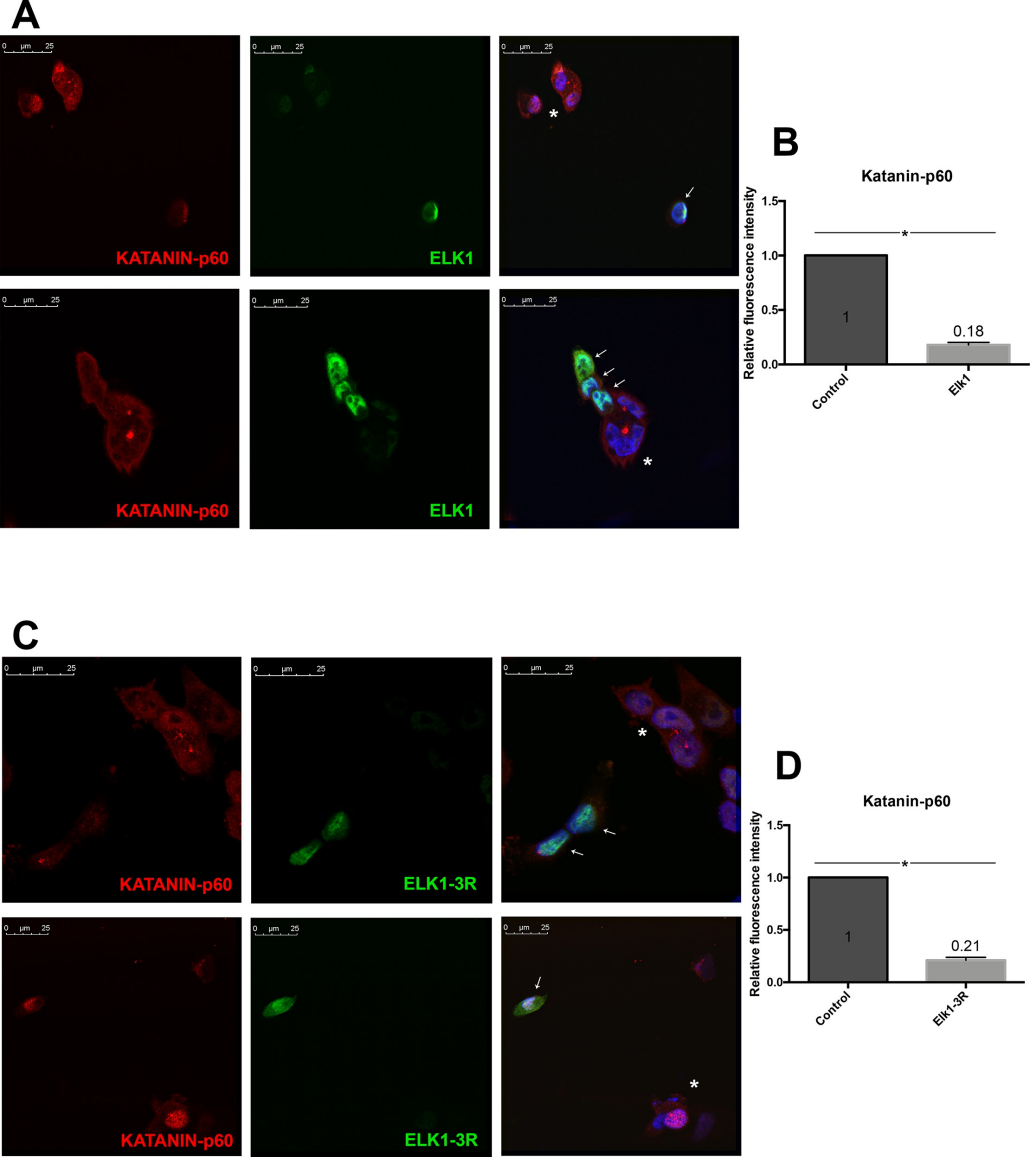
ICC results of Elk1 and Elk1-3R effects on endogenous katanin-p60 level. Asterisk symbol represents untransfected cells, whereas the cells indicated with arrows are either Elk1 or Elk1-3R overexpressing cells. The images were taken using 63X objective at zoom 2.6. (A) The level of endogenous katanin-p60 protein is reduced in Elk1 overexpressed SH-SY5Y cells compared to untransfected cells. (B) Relative fluorescence density graph shows that the expression of katanin-p60 was 5.5 fold decreased in Elk1 overexpressed cells compared to control cells. SEM values are 0 and 0.02, respectively. (C) The decrease in endogenous katanin-p60 level is observed in Elk1-3R overexpressed SH-SY5Y cells compared to untransfected cells. (D) Relative fluorescence density graph shows that the expression of katanin-p60 was 4.76 fold decreased in Elk1-3R overexpressed cells compared to control cells. SEM values are 0 and 0.03, respectively.

**Fig 8:**
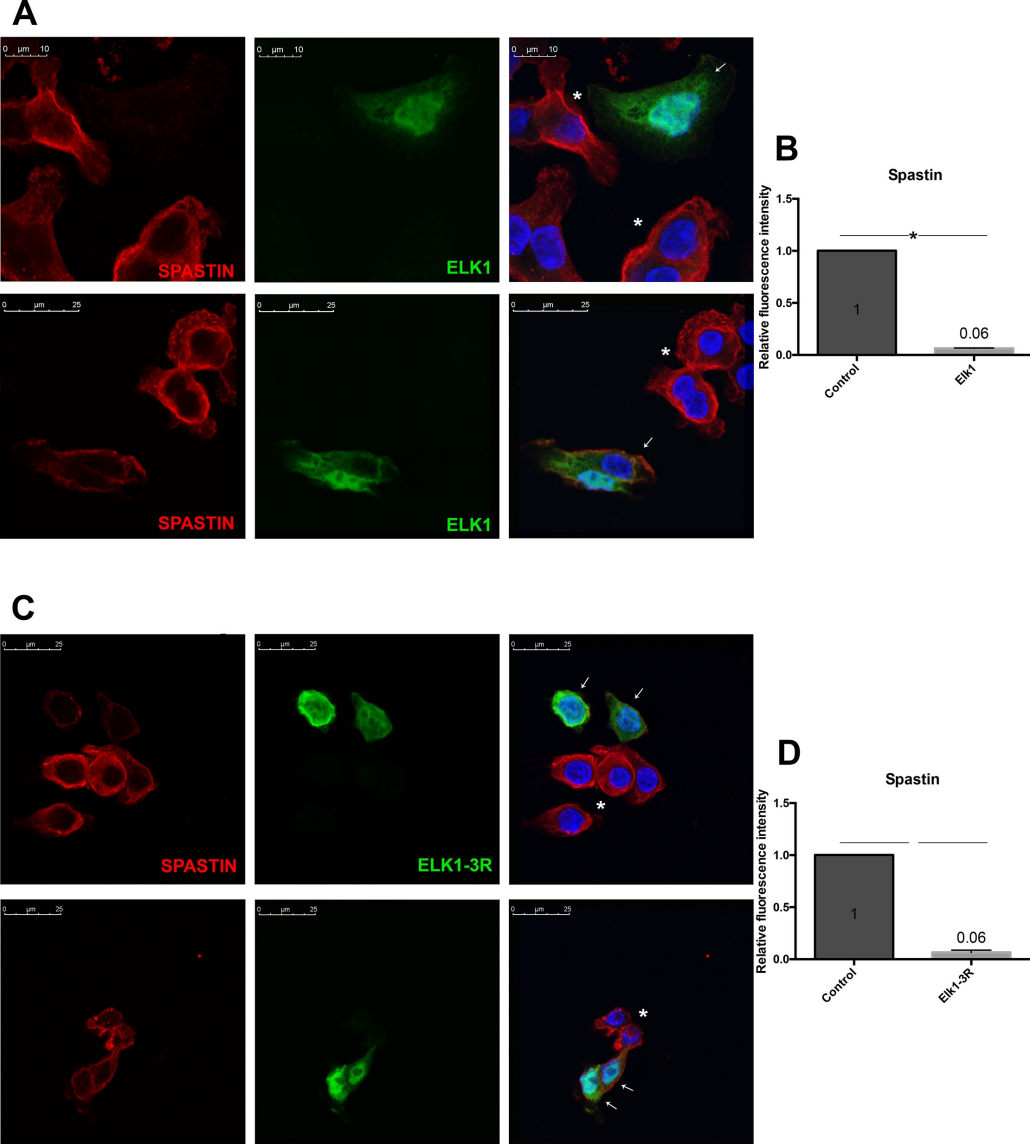
ICC results of Elk1 and Elk1-3R effects on endogenous spastin level. Asterisk symbol represents untransfected cells, whereas the cells indicated with arrows are either Elk1 or Elk1-3R overexpressing cells. The images were taken using 63X objective at zoom 2.6. (A) In comparison to untransfected cells, Elk1 overexpression in SH-SY5Y cells leads to decreased endogenous spastin protein level. (B) Relative fluorescence density graph shows that the expression of spastin was 16.6 fold decreased in Elk1 overexpressed cells compared to control cells. SEM values are 0 and 0.003, respectively. (C) Elk1-3R overexpression in SH-SY5Y cells results in reduced endogenous spastin level compared to untransfected cells. (D) Relative fluorescence density graph shows that the expression of spastin was 16.6 fold decreased in Elk1-3R overexpressed cells compared to control cells. SEM values are 0 and 0.02, respectively.

The repressor effect was also detected by performing ICC via nucleofection of both constructs. Elk1 or Elk1-3R nucleofected cells were visualized by staining with either FLAG-tag or His-tag antibody since pCMV6 vectors include both FLAG-tag and His-tag. The level of katanin-p60 was reduced in both Elk1 (Fig 7A) and Elk1-3R (Fig 7C) overexpressing cells in comparison with non-overexpressing cells. The graphs (Fig 7B and 7D) showed that katanin-p60 expression was decreased in 5.5 fold in Elk1 overexpresed cells compared to control cells and 4.76 fold in Elk1-3R overexpressed cells compared to control cells. The decrease in both conditions was found as significant according to the statistical analyses. The same results were observed in spastin level upon Elk1 (Fig 8A) or Elk1-3R (Fig 8C) overexpression. According to the graphs (Fig 8B and 8D), spastin expression was decreased in 16.6 fold in both Elk1 overexpressed and Elk1-3R overexpressed cells compared to control cells. The decrease in both cells were found as significant by statistical analyses. In addition, both Elk1 and Elk1-3R were localized in both nucleus and cytoplasm.

### Three lysine mutations (K230R, K249R, K254R) are not enough to prevent Elk1 SUMOylation

**Fig 9:**
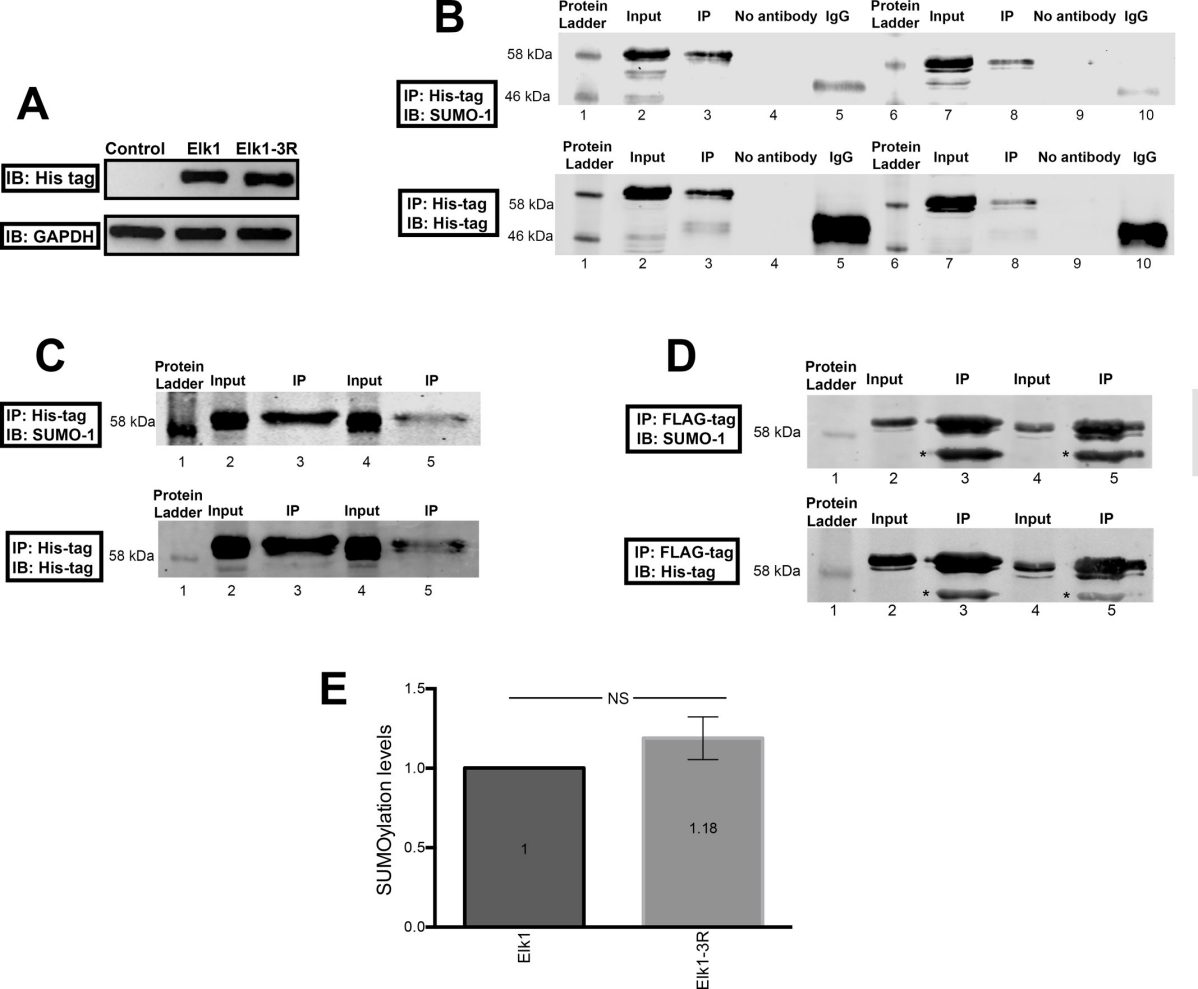
Immunoprecipitation results for SUMOylation of Elk1 and Elk1-3R. (A) Before IP experiments, transfection of SH-SY5Y cells treated with KCl was verified via Western blotting targeting His-tag of the vectors. Upper part of the figure shows the overexpression of both Elk1 and Elk1-3R, while the expression is not detected in untransfected control cells. Lower part of the figure represents GAPDH expression which was used as loading control for each reaction. (B) SUMO-1 detection (upper part) and immunoprecipitation control (lower part) were shown in the result of His-tag antibody used IP experiment. Lane 1 and 6: Protein Standard; Lane 2: wt-Elk1 input; Lane 3: wt-Elk1 IP; Lane 4: wt-Elk1 no antibody; Lane 5: wt-Elk IgG; Lane 7: Elk1-3R input, Lane 8: Elk1-3R IP, Lane 9: Elk1-3R no antibody; Lane 10: Elk1-3R IgG. (C) The result of IP experiment performed by using nickel particles indicates SUMO-1 detection (upper part) and immunoprecipitation control (lower part). Lane 1: Protein Standard, Lane 2: wt-Elk1 input, Lane 3:wt-Elk1 IP, Lane 4: Elk1-3R input, Lane 5: Elk1-3R IP. (D) FLAG-tag resin used IP result exhibits SUMO-1 detection (upper part) and immunoprecipitation control (lower part). Lane 1: Protein Standard, Lane 2: wt-Elk1 input, Lane 3:wt-Elk1 IP, Lane 4: Elk1-3R input, Lane 5: Elk1-3R IP. The * symbol represents the heavy chain of IgG. (E) The graph of normalized SUMOylation levels to precipitations followed by normalization of Elk1-3R to Elk1. The result shows the increase in Elk1-3R SUMOylation compared to Elk1, however, the increase is not significant. SEM values are 0 and 0,13, respectively.

The difference between SUMOylation levels of Elk1 and Elk1-3R was evaluated by IP experiments using different methods in order to ensure the results. To verify expression upon transfection, Elk1 or Elk1-3R (both conjugated to His-tag and FLAG-tag) expression was confirmed using His-tag antibody (Fig 9A). Then, IP was performed using Elk1 and Elk1-3R overexpressed cell lysates. In order to distinguish the SUMOylation of endogenous Elk1 from overexpressed Elk1 and Elk1-3R, either His-tag or FLAG-tag of exogenously expressed Elk1 and Elk1-3R were targeted for precipitations. The result of the first experiment, which was performed using His-tag antibody, is given in Fig 9B. Immunoprecipitated Elk1 (lane 3) and Elk1-3R (lane 8) SUMOylations are shown in upper part of Fig 9B. Also, the precipitation controls of Elk1 (lane 3) and Elk1-3R (lane 8) using His-tag antibody are indicated in lower part of Fig 9B.

Since both Elk1 and Elk1-3R contain His-tag, IP experiment was repeated for confirmation using nickel particles that have a strong affinity to His-tag (Fig 9C). SUMOylation of both proteins was detected in lane 3 and lane 5 which represent precipitated Elk1 and Elk1-3R, respectively (upper part of Fig 9C). The precipitation of Elk1 and Elk1-3R were detected using His-tag antibody (lower part of Fig 9C).

Finally, the result of IP experiment for another confirmation using FLAG-tag resin is given in Fig 9D. SUMO-1 detection (upper part of Fig 9D) and precipitation control using His-tag antibody (lower part of Fig 9D) confirmed the previous experiments and concluded that both proteins are able to be SUMOylated. Also, no visible difference between SUMOylation levels of Elk1 and Elk1-3R was detected. Normalized SUMOylation levels of both Elk1 and Elk1-3R were indicated in Fig 9E. Even though there was a slight difference between the levels in which Elk1-3R was shown as to be more SUMOylated, the difference was not significant according to the statistical analysis.

### Methylation alters Elk1 binding to *KATNA1*, but not to *SPG4*

**Fig 10:**
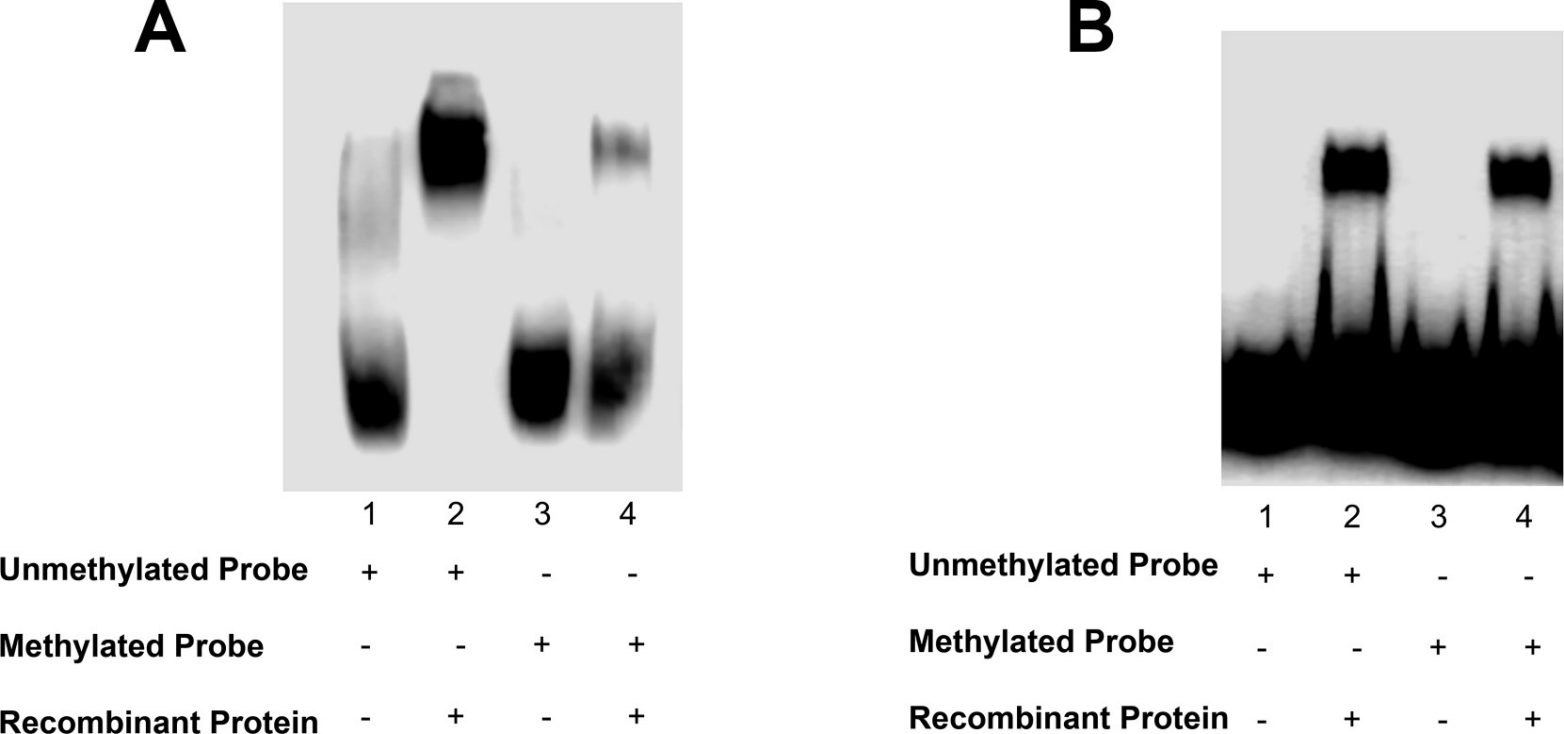
The effect of methylation on Elk1 binding. (A) Unmethylated biotin-labeled WT oligonucleotide including +113/+122 Elk1 binding site on *KATNA1* is shown in lane 1. When +113/+122 Elk1 binding site is incubated with Elk1-db protein, shift band is observed (lane 2). This oligonucleotide was methylated and loaded as control (lane 3). In the presence of Elk1-db protein and methylated oligonucleotide, the shift band intensity (lane 4) is clearly lower compared to unmethylated oligo (lane 2) (n = 3). (B) Elk1 binding is observed when incubated with unmethylated *SPG4* oligonucleotide including -235/-226 binding site (lane 2). Also, methylated form of this oligonucleotide could not prevent the complex formation with Elk-1 (lane 4). Both unmethylated and methylated probes were separately loaded as controls (lane 1 and 3, respectively) (n = 3).

Since methylation has a key role for transcriptional regulation, we analyzed CpG islands on *KATNA1* and *SPG4* (Fig 1A) sequences via EBI, EMBOSS CpGPlot, and the binding sites of Elk1 are contained on the CpG islands according to the analyses. Thus, we performed EMSA using methylated and unmethylated probes which contain +113/+122 binding site of *KATNA1*, and -451/-442 and -235/-226 binding sites of *SPG4* (Table 6) in order to evaluate whether methylation could prevent the interaction with Elk1. The results clearly showed that methylation inhibits Elk1 binding to *KATNA1* (Fig 10A). Band intensity of methylated probe shift was very low compared to unmethylated probe shift. However, no difference was detected between unmethylated and methylated probe shift of *SPG4* (Fig 10B). In conclusion, methylation did not affect the binding ability of Elk1 to *SPG4*.

## Discussion

Initiation of transcription requires the addition of RNA polymerase II to preinitiation complex (PIC) at the core promoter, which is defined as the region from -40 bp upstream to +40 bp downstream of transcription start site of genes. TATA-box and Initiator have been shown to play key roles in accurate positioning of the transcription start site. However, most of the characterized eukaryotic gene promoters have been shown to lack these two cis-acting elements. Assembly of PIC on such promoters is likely to be regulated alternatively by other regulatory elements. In TATA-less promoters; M3, M6, and M22 motifs which respectively correspond to Elk-1 (SCGGAAGY), Sp1 (GGGCGGR) and non-category (TGCGCANK) binding sites have been found to be enriched [43]. These findings suggest that Elk-1 might have a prominent regulatory role in transcription initiation of TATA-less promoters.

In this study, we initially determined the critical region of *KATNA1*, which lacks TATA-box and Initiator at the promoter site. To do this, we generated four deletion constructs including “promoter”, “5’ UTR”, “promoter + 5’ UTR”, “promoter + 5’ UTR + intron” sequences (upper part of Fig 1A). We showed that the promoter did not have a regulatory effect on *KATNA1* expression, whereas 5’ UTR displayed the highest activity (Fig 1B). The reason for this might be the presence of TATA-less promoter motifs in the 5’ UTR of *KATNA1* as described earlier. Also, the repressive effect of the intron region of *KATNA1* could be due to other transcription factors’ inhibitory effects or the presence of silencer regions.

Since Elk1 has been determined as a neuronal transcription factor due to its abundant expression in human brain and participation in different neuronal processes, such as neuronal differentiation and viability, it could have a key role in the regulation of microtubule severing proteins; katanin and spastin which are critical for neuronal differentiation [31,32,44]. Moreover, katanin-p80 (*KATNB1*) and spastin (*SPG4*) regulations by Elk1 have been previously identified [36,37].

In this study, Elk1 binding to *KATNA1* was investigated in detail. In order to identify Elk1 binding sites on *KATNA1* theoretically, two bioinformatic tools were used, however they indicated different results. PROMO tool [39,40] predicted three binding sites (upper part of Fig 1A). One of them is located on the promoter (-428 to -419), whereas the other two sites are on the 5’ UTR (+113 to +122 and +253 to +262), and these binding sites have 0.13%, 8.90%, and 2.16% dissimilarity rates, respectively. However, MATCH tool [41] indicated only one site located from +113 to +122. We also compared these sequences to Elk1 consensus motif (CCGGAAGT), which was obtained from JASPAR database [45], and found that only +113/+122 site exactly matched to the consensus sequence. Yet, all of the three regions were investigated to reveal which site or sites are functional for Elk1 binding. The results indicated that Elk1 binds to only +113/+122 site of *KATNA1* which was correctly predicted by MATCH tool (Figs 2 and 3). Therefore, MATCH tool seemed to be more efficient at determining functionally active binding sites for transcription factors. Besides *KATNA1*, we had determined Elk1 binding sites on *SPG4* as -451/-442 and -235/-226 in our previous study [36], both of which are located on the promoter region and not on the 5’ UTR (Fig 1A).

The effect of Elk1 on *KATNA1* and *SPG4* transcriptional regulation was investigated and it was revealed that Elk1 acted as a transcriptional activator of both genes (Fig 4). However, upon Elk1 overexpression, both *KATNA1* and *SPG4* reporter protein levels and also endogenous katanin-p60 and spastin protein levels were decreased, as confirmed in luciferase reporter assay (Fig 5), Western blotting (Fig 6), and ICC experiments (Figs 7A and 8A). The contradictory results on mRNA and protein levels implied the presence of a post-transcriptional regulation which could be arisen from either miRNA-mediated regulation or RNA binding proteins (RBPs). It is known that two different mechanisms exist for miRNA-mediated regulation; translational repression and mRNA decay [46]. However, mRNA decay could not elucidate *KATNA1* and *SPG4* regulation as it would result in mRNA degradation, whereas both *KATNA1* and *SPG4* mRNA levels were increased. Additionally, RBPs are also involved in miRNA-mediated translational repression. These findings led us to investigate which RBPs could possibly participate in *KATNA1* and *SPG4* regulation by Elk1. Bioinformatic analysis using CLIPdb tool [47,48] (http://lulab.life.tsinghua.edu.cn/clipdb/index.php) indicates 16 RBPs for *KATNA1* and 25 RBPs for *SPG4*. 12 RBPs target both genes. Among these, due to being a member of ELAV-like (Hu) protein family, which is involved in neuronal development and differentiation, neurological disorders, and neuron-specific expressions [49]; HuR was chosen to be analyzed for its effect on post-transcriptional regulations of *KATNA1* and *SPG4*. Also, it was previously suggested that HuR is able to enhance translational repression due to binding to cis-acting elements present in the 5’ UTR of p27 encoded by *CDKN1B* gene, therefore HuR is an important factor to control cell proliferation by decreasing the level of cell cycle inhibitor [50,51].

In order to understand if the decrease in katanin-p60 and spastin protein levels could possibly be mediated by HuR, we first investigated p27 encoding *CDKN1B* mRNA and p27 protein levels in Elk1 and Elk1-3R overexpressed cells. The results showed that *CDKN1B* mRNA level increased, while p27 protein level decreased in Elk1 and Elk1-3R transfected cells (S2 and S3 Figs). Then, HuR level was investigated as it was previously shown that increased HuR protein caused a decrease in p27 protein level [50]. We also found that HuR was significantly increased as a result of Elk1 and Elk1-3R overexpression (S4 Fig). Thus, it could be suggested that the increased HuR levels may promote translational repression of both *KATNA1* and *SPG4*. Moreover, the known HuR binding motifs; AUUUA, AUUUUA, AUUUUUA, and UUUCCUU are repetitively present in *KATNA1* and *SPG4* mRNAs, whereas *KATNB1* mRNA has a very few of these binding sites [52,53]. This finding might explain the consistent results between mRNA and protein levels of *KATNB1* encoding katanin-p80, both of which increased upon Elk1 overexpression [37].

Elk1 leads to up-regulation of katanin-p80 [37] and down-regulation of spastin [36] and katanin-p60. Based on the opposite findings on katanin p60 and p80 subunits; it might be suggested that Elk1 results in stabilization of microtubules by restricting the severing activity due to its repressive effect on katanin-p60. Elk1 could also be suggested to play a role in centrosome targeting of katanin-p60 by increasing katanin-p80; thereby, restricting the formation of non-centrosomal microtubules in neurons. Non-centrosomal microtubule formation is required to increase neuronal branching and neurite formation in neurons [54], and this process requires increased microtubule severing by activation of katanin-p60, which is inhibited upon overexpression of Elk. Also, it has been known that overexpression of katanin-p60 is deleterious for axonal growth due to excessively reducing microtubule mass and katanin-p80 might undertake a control mechanism which guides the degree of microtubule severing [8,9]. Thus, Elk1 might contribute to this control mechanism by decreasing katanin-p60 and increasing katanin-p80 expressions.

Other microtubule severing enzymes like fidgetin and VPS4 have also been shown to be important for axonal growh and development of the nervous system. Whether or not Elk1 might contribute to regulation of other microtubule severing proteins’control mechanisms physiologically remains to be investigated However, when we searched possible transcriptional regulation theoretically we identified putative binding and possible regulation sites of Elk1 on fidgetin and VPS4 which are awating for functional confirmation. In our theoretical search, MATCH tool did not indicate any binding site of Elk1 on *FIGN* promoter and 5’ UTR, whereas PROMO tool predicted one binding site from -523 to -531 on *FIGN* promoter when the maximum dissimilarity rate was adjusted as 9%. Likewise *VSP4A* and *VSP4B* do not include any Elk1 binding site according to MATCH tool, while PROMO asserted several binding sites which were from +37 to +45 on 5’ UTR of *VSP4A* and from -982 to -990, from -753 to -761, from -179 to -187 and from -96 to -104 on promoter of *VSP4B*. Yet, based on our experience as MATCH tool was found out to be more efficient at determining physiologically relevant binding sites for transcription factors and given that *FIGN* and *VSP4* promoter and UTR regions do not indicate any binding, Elk1 might not be the functional transcription factor for these genes and its regulation could be specific for spastin and katanin proteins.

Elk1 has been described as having pro-differentiation function within the nucleus of neurons [55]. However, the only well-known direct mechanism of Elk1 in the regulation of neuronal differentiation is alteration of *pip92* gene expression which is an IEG. As a transcription factor, Elk1 binds to the upstream promoter region of *pip92* and induces its expression that results in negative modulation of neuronal differentiation [31]. On the other hand; based on opposing effects of Elk1 on katanin-p60 and katanin p80, Elk1 could also play a role in neuronal differentiation process through alteration of microtubule organization by decreasing katanin-p60. Therefore, Elk1-related neuronal differentiation mechanism needs to be further investigated in neuronal cells.

It has been revealed that in addition to ETS (A) domain, R domain of Elk1 has a repressor activity. The mechanism is presumably related to SUMO modifications on the R domain which promote the recruitment of Histone Deacetylases (HDAC) and transcriptional repression since HDACs keep the promoters in deacetylated and inactive state [56,57]. However, HDAC-ETS domain interaction could be destroyed by ERK MAPK activation which phosphorylates serine residues at the C domain. Consequently, SUMOylation and phosphorylation could affect Elk1 activity [58]. In our previous study, we demonstrated that Elk1 causes repression of spastin expression and speculated that it might be related to SUMOylation. For this purpose, we first generated Elk1-3R construct by converting three lysine residues at the R domain into arginine (K230R, K249R, K254R), which were indicated as the main SUMO modification sites [26]. The confirmation of these mutations by Sanger sequencing (S5 Fig) was followed by the investigation of Elk1-3R effect on *KATNA1* and *SPG4* transcriptional regulation and protein expression. We observed that Elk1-3R had an activator role in transcription (Fig 4), whereas both endogenous and reporter protein levels were reduced in the presence of Elk1-3R (Figs 5, 6, 7B and 8B). Also, we did not observe a statistically meaningful difference between Elk1 and Elk1-3R on *KATNA1* and *SPG4* transcriptional and translational expressions. Firstly, we attributed these similar effects of Elk1 and Elk1-3R to two reasons: One of them was that the elimination of Elk1 SUMOylation in the R domain could not lead to any alteration due to the promoter-specific effect of SUMOylation [25]. Another reason was associated with ETS (A) domain which has a stronger repressor effect than the R domain [58].

In the study by Salinas *et al*. [26] the SUMOylation has been claimed to regulate nucleo-cytoplasmic shuttling, and Elk1-3R is expected to be localized in the nucleus. Yet, they showed no difference between wt-Elk1 and Elk1-3R in terms of cellular localization in HeLa cells. However, they suggested that Elk1-3R relocalizes to the nucleus more rapidly than wt-Elk1 in HeLa/Balb/C heterokaryons. We also obtained similar results for the localization of Elk1 and Elk1-3R in SH-SY5Y cells (Figs 7 and 8), and our results led us to investigate SUMOylation levels of both Elk1 and Elk1-3R proteins. Even though four SUMO isoforms are available in mammalian cells (SUMO-1, SUMO-2, SUMO-3, and SUMO-4), SUMO-1 was chosen for investigation since SUMOylation of Elk1 is predominantly conducted by SUMO-1 [37] and SUMO-1 exists as a complex with its target proteins in the cell, while SUMO-2/3 appears in the free form and conjugates to substrates as a cellular response to environmental stresses like temperature fluctuation [29]. Additionally, SUMO-4 was not chosen because of the restriction of its expression to renal, immune, and pancreatic cells and also human placenta [59]. Surprisingly, our results showed that Elk1-3R, which was previously indicated to be a SUMO mutant by Salinas *et al*. [26], could also be SUMOylated like wt-Elk1. Since this was a surprising result, we confirmed this finding using different methods and the results indicating SUMOylation of Elk1-3R were consistent in each experiment (Fig 9). The canonical conserved motif of SUMOylation has been accepted as ѱKxE or ѱKxE/D. However, the presence of non-canonical or non-conserved motifs has also been demonstrated [60,61]. In addition, it has been also revealed that there are some SUMO-interaction motifs (SIMs) where SUMO binds target protein by a non-covalent bond as distinct from SUMO-conjugation sites which allow covalent binding of SUMO [62–65]. The reason of Elk1-3R SUMOylation might be related to other sites such as non-canonical motifs or SIMs. Moreover, some possible SIMs between 364–367, and 418–421 residues and non-consensus motifs such as K35, K130, and K271 of Elk1 were also detected through bioinformatic analysis. It is possible that these SIMs and non-consensus motifs could have roles in SUMOylation of Elk1. Yet, post-translational modifications of Elk1, which are SUMOylation and phosphorylation, have an impact on its functionality in different cellular locations [55]. For instance, Elk1 overexpression in distal dendrites has been identified as toxic for neurons, while the overexpression in cell body has not any effect on neuronal death. It has been assumed that those functions have arisen from post-translational modifications [32]. Phosphorylation of Elk1 has been also associated with some pathophysiological conditions, such as Alzheimer, Huntington, Down syndrome, synucleinopathies and depression [55]. In addition to the effect of post-translational modifications of Elk1 on those conditions, katanin-p60 effect based on Elk1 regulation needs to be further investigated.

Due to the fact that human TATA-less promoters have high GC content [43], we searched for CpG islands using EBI, EMBOSS CpGPlot/Report tool. It was revealed that *KATNA1* and *SPG4* have CpG islands located between -85/+218 and -566/+589, respectively. These CpG islands also contain Elk1 binding sites (Fig 1A). In regard to the bioinformatic analysis, it was thought that methylation might be an important mechanism for regulation of both genes. The results showed that *KATNA1* methylation inhibited Elk1 binding on +113/+122 site (Fig 10A). However, there was no difference in the binding ability of Elk1 to *SPG4* at both -235/-226 (Fig 10B). The reason of these findings is presumably related to CpG dinucleotide number and location. *KATNA1* oligonucleotide has five CpG dinucleotides and one of them is present within the Elk1 binding site (Table 6). However, one of the *SPG4* oligonucleotide (WT_1) has one CpG dinucleotide which is located outside of the binding site. Even though other *SPG4* oligonucleotide (WT_2) has one more CpG dinucleotide, it is not sufficient to prevent the interaction; probably, as it resides right at the start of the binding site (Table 6).

Elk1 binding site on *KATNA1* has a highly conserved sequence according to the UCSC Genome Browser (S1 Fig), therefore methylation might have a key role in transcriptional regulation. However, it may not be an important mechanism for *SPG4* regulation in the view of the fact that Elk1 binding site on *SPG4* has a poorly conserved sequence [66].

In conclusion, we identified the critical region on *KATNA1* promoter and showed that *KATNA1* gene expression is regulated by Elk1 via RBPs and methylation-dependent mechanism. This study revealed a mechanism to understand different gene expression patterns of katanin-p60 and katanin-p80 proteins resulting in different katanin-p60/katanin-p80 ratios both in development and different tissues, causing different degree of severing by katanin-p60. The role of epigenetic mechanisms in the regulation of microtubule severing awaits for further investigation.

## Supporting information

S1 Fig Conservation of Elk1 binding sites among species. Elk1 binding site located at position +113/+122 (5’ UTR) is highly conserved among species. The upper pane (above dashed line) shows conservation track of *KATNA1* promoter (500 bp) and 5’ UTR (349 bp). Bioinformatically identified Elk1 binding sites on promoter and 5’ UTR are indicated by vertical double red lines. Lower panel shows zoomed alignment of each Elk1 binding sites. Elk1 binding sequences are indicated by red rectangles. Blue peaks indicate basewise conservation by PhyloP, Green peaks indicate conservation by PhasCons and claret red bars indicate conserved elements among 100 vertebrate species.
(PDF)

S2 Fig The level of *CDKN1B* (p27) mRNA. Both Elk1 and Elk1-3R significantly increased *CDKN1B* (p27) mRNA level compared to control. SEM values are 0, 0.05, and 0.02, respectively. Each experiment was performed as triplicates on the same day and the experiments were repeated three times on separate days, independently (n = 3).
(TIF)

S3 Fig The level of p27 protein. (A) Western blotting results of p27 protein. Lane 1: untransfected control cell lysate, Lane 2: Elk1 overexpressed cell lysate, Lane 3: Elk1-3R overexpressed cell lysate. Upper part of the figure shows reduced p27 levels in Elk1 and Elk1-3R overexpressed cells compared to control cells. Lower part indicates vinculin expression as loading control. (B) Quantification result of normalized p27 protein level. Both Elk1 and Elk1-3R significantly decreased the amount of p27 protein. SEM values are 0, 0.08, and 0.06, respectively. Transfection and Western blotting experiments were repeated three times on separate days, independently (n = 3).
(TIF)

S4 Fig The level of HuR protein. (A) Western blotting results of HuR. Lane 1: untransfected control cell lysate, Lane 2: Elk1 overexpressed cell lysate, Lane 3: Elk1-3R overexpressed cell lysate. In comparison to control cells, both Elk1 and Elk1-3R overexpression result in increased HuR level indicated in upper part of the figure. Lower part shows vinculin expression as loading control. (B) Quantification result of normalized HuR level. HuR level was significantly increased in Elk1 and Elk1-3R overexpressed cell lysates. SEM values are 0, 0.11, and 0.14, respectively. Transfection and Western blotting experiments were repeated three times on separate days, independently (n = 3).
(TIF)

S5 Fig Sanger sequencing chromatograms of site-directed mutageneses. Upper part of the figure represents Elk1 chromatograms, while lower part indicates Elk1-3R. Lysine amino acids located on 230 (A), 249 (B), 254 (C) residues were converted into arginines to generate Elk1-3R construct.
(TIF)

S1 Data Raw data of Fig 2A. (TIF)

S2 Data Raw data of Fig 2B. (TIF)

S3 Data Raw data of Fig 2C. (TIF)

S4 Data Raw data of Fig 3A. Last 2 wells includes irrelevant samples, thus, they were denoted by vertical black line by using Adobe Photoshop CS6 software.
(TIF)

S5 Data Raw data of Fig 3B. (TIF)

S6 Data Raw data of Fig 3C. Last well includes irrelevant sample, thus, it was denoted by vertical black line by using Adobe Photoshop CS6 software.
(TIF)

S7 Data Raw data of upper part of Fig 4A. Color Prestained Protein Standard, Broad Range (NEB) was used as molecular marker.
(TIF)

S8 Data Raw data of lower part of Fig 4A. Color Prestained Protein Standard, Broad Range (NEB) was used as molecular marker.
(TIF)

S9 Data Raw data of upper part of Fig 6B. The wells on the left side of molecular marker includes irrelevant lysates of another cell type, however, they had been used in order to reveal whether non-specific bands were still present in those samples. Since the antibody was polyclonal, non-specific bands were obtained in each experiment. However, single band in each well was located in between 80 kDa and 58 kDa as near to 58 kDa, also, it had the most strong intensity. Since katanin-p60 is expected as 60 kDa, the band mentioned above was chosen unhesitantly. Color Prestained Protein Standard, Broad Range (NEB) was used as molecular marker. The wells on the left site of molecular marker were denoted by vertical black line by using Adobe Photoshop CS6 software.
(TIF)

S10 Data Raw data of lower part of Fig 6B. (TIF)

S11 Data Raw data of upper part of Fig 6D. Even though the wells on the left side of molecular marker included same samples, they were denoted by vertical line by using Adobe Photoshop CS6 software, as they were not represented in main figure. The bands which were present under our specific bands could be another isoform of spastin instead of non-specific bands, since their level were also decreased. Color Prestained Protein Standard, Broad Range (NEB) was used as molecular marker.
(TIF)

S12 Data Raw data of lower part of Fig 6D. Even though the wells on the left side of molecular marker included same samples, they were denoted by vertical line by using Adobe Photoshop CS6 software, as they were not represented in the main figure. Color Prestained Protein Standard, Broad Range (NEB) was used as molecular marker.
(TIF)

S13 Data Raw data of upper part of Fig 9A. The wells on the right side were denoted by vertical line by using Adobe Photoshop CS6, since they were irrelevant to the our experiment. Color Prestained Protein Standard, Broad Range (NEB) was used as molecular marker.
(TIF)

S14 Data Raw data of lower part of Fig 9A. The wells on the right side were denoted by vertical line by using Adobe Photoshop CS6, since they were irrelevant to our experiment. Color Prestained Protein Standard, Broad Range (NEB) was used as molecular marker.
(TIF)

S15 Data Raw data of upper part of Fig 9B. Color Prestained Protein Standard, Broad Range (NEB) was used as molecular marker.
(TIF)

S16 Data Raw data of lower part of Fig 9B. Color Prestained Protein Standard, Broad Range (NEB) was used as molecular marker.
(TIF)

S17 Data Raw data of upper part of Fig 9C and 9D. Color Prestained Protein Standard, Broad Range (NEB) was used as molecular marker.
(TIF)

S18 Data Raw data of lower part of Fig 9C and 9D. Color Prestained Protein Standard, Broad Range (NEB) was used as molecular marker.
(TIF)

S19 Data Raw data of Fig 10A. The first 4 wells were denoted by vertical line by using Adobe Photoshop CS6, since they included irrelevant samples.
(TIF)

S20 Data Raw data of Fig 10B. Since first 5 wells included irrelevant samples and the last well included higher probe concentration than other wells, they were denoted by vertical lines by using Adobe Photoshop CS6. The main figure in manuscript has been represented in between the lines.
(TIF)
