# Supplementary figures and images for: Elk1 affects katanin and spastin proteins via differential transcriptional and post-transcriptional regulations
Source: PLoS One. 2019 Feb 21;14(2):e0212518. doi: 10.1371/journal.pone.0212518 (PMC6383945; doi:10.1371/journal.pone.0212518)

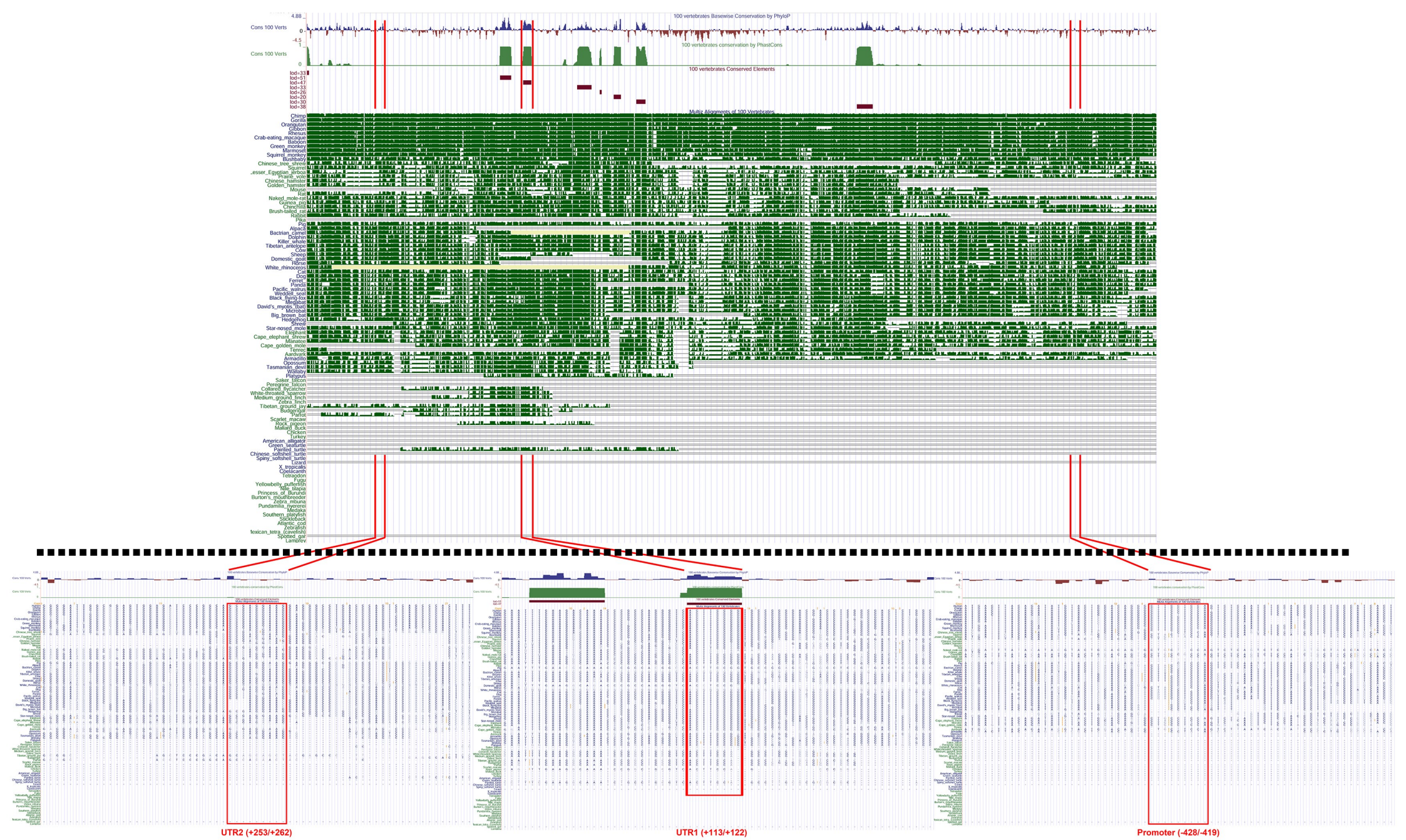

Supplement: S1 Fig — Elk1 binding site located at position +113/+122 (5’ UTR) is highly conserved among species. The upper pane (above dashed line) shows conservation track of KATNA1 promoter (500 bp) and 5’ UTR (349 bp). Bioinformatically identified Elk1 binding sites on promoter and 5’ UTR are indicated by vertical double red lines. Lower panel shows zoomed alignment of each Elk1 binding sites. Elk1 binding sequences are indicated by red rectangles. Blue peaks indicate basewise conservation by PhyloP, Green peaks indicate conservation by PhasCons and claret red bars indicate conserved elements among 100 vertebrate species. (PDF) [file pone.0212518.s001.pdf]

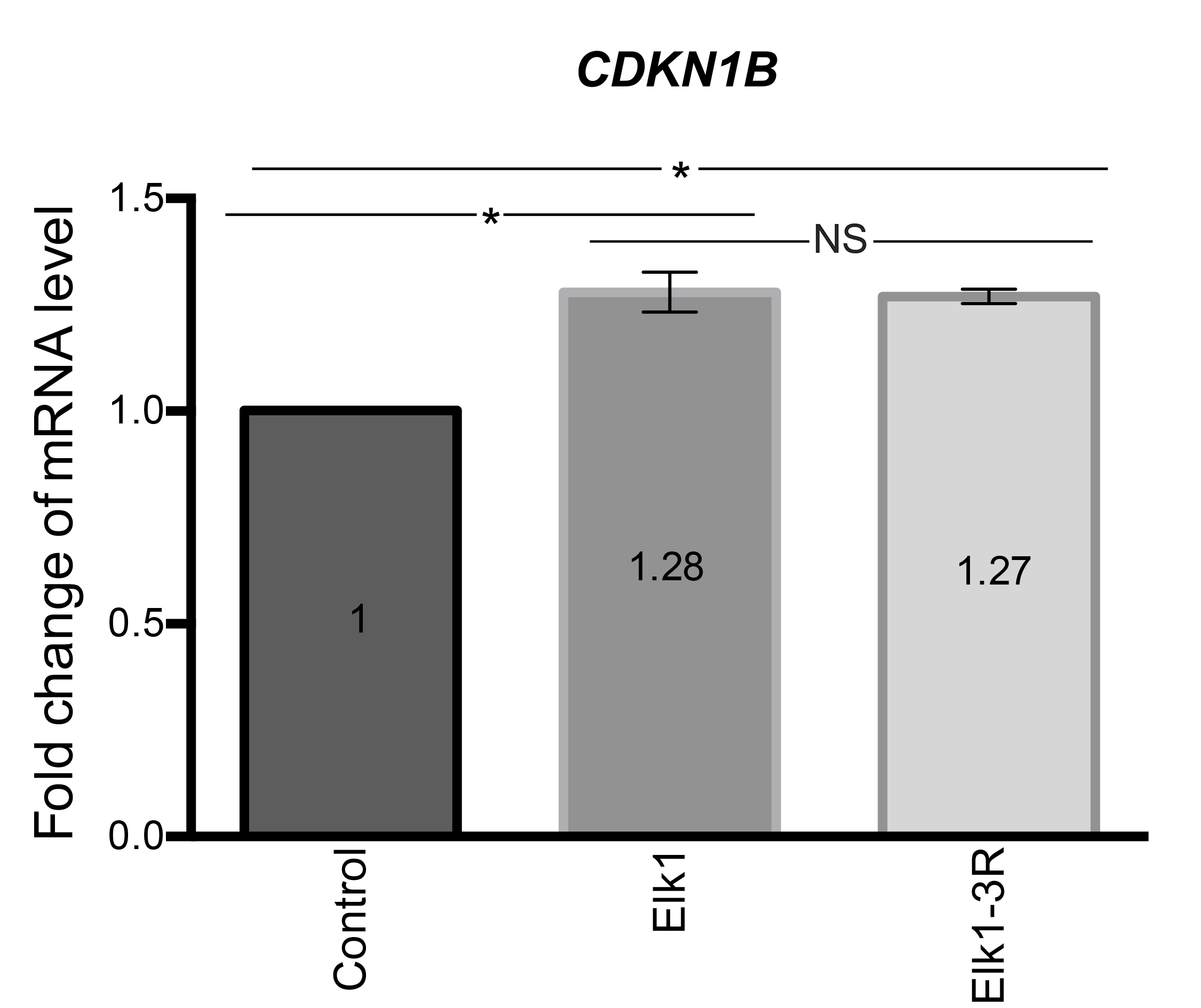

Supplement: S2 Fig — Both Elk1 and Elk1-3R significantly increased CDKN1B (p27) mRNA level compared to control. SEM values are 0, 0.05, and 0.02, respectively. Each experiment was performed as triplicates on the same day and the experiments were repeated three times on separate days, independently (n = 3). (TIF) [file pone.0212518.s002.tif]

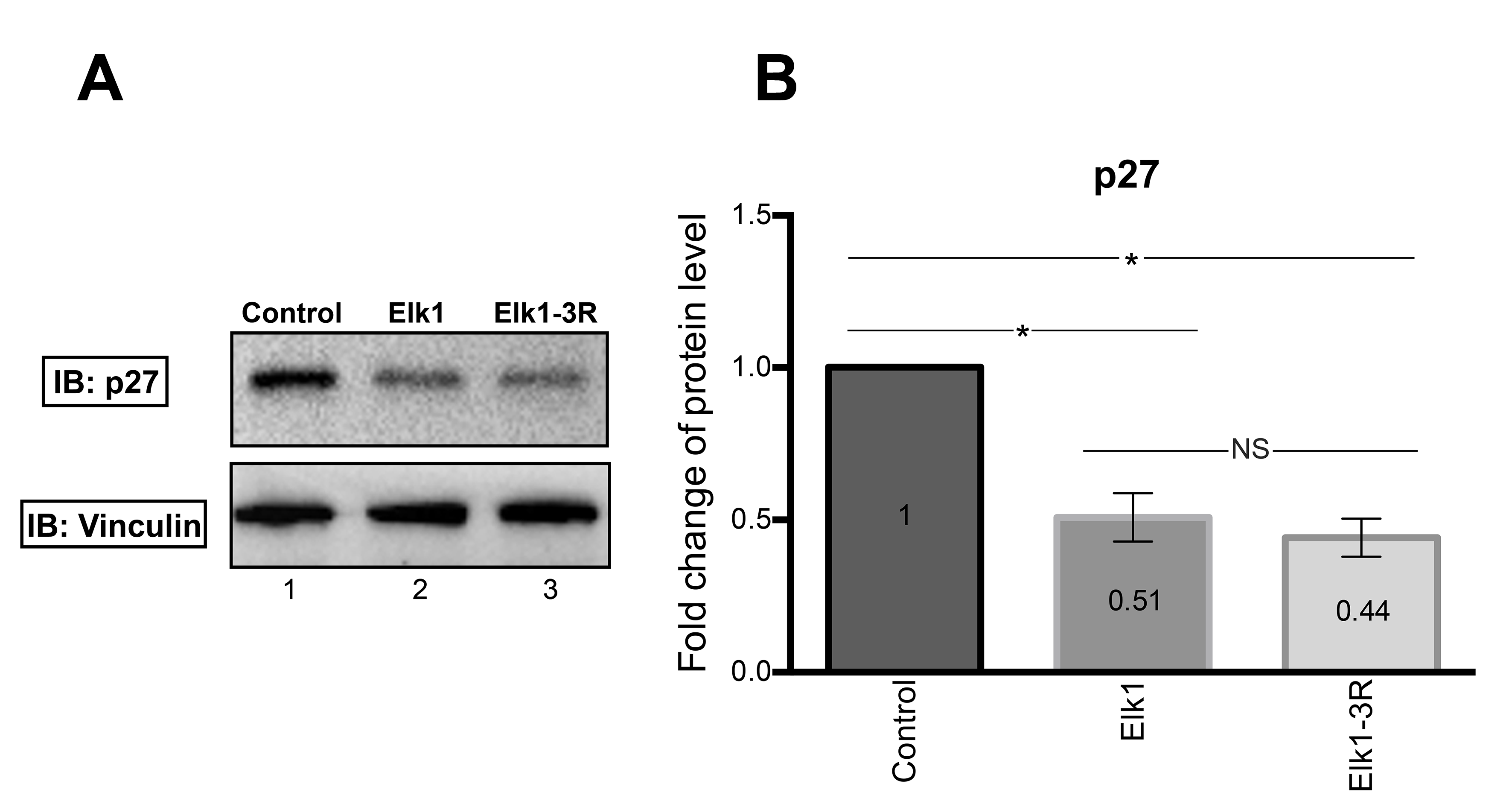

Supplement: S3 Fig — (A) Western blotting results of p27 protein. Lane 1: untransfected control cell lysate, Lane 2: Elk1 overexpressed cell lysate, Lane 3: Elk1-3R overexpressed cell lysate. Upper part of the figure shows reduced p27 levels in Elk1 and Elk1-3R overexpressed cells compared to control cells. Lower part indicates vinculin expression as loading control. (B) Quantification result of normalized p27 protein level. Both Elk1 and Elk1-3R significantly decreased the amount of p27 protein. SEM values are 0, 0.08, and 0.06, respectively. Transfection and Western blotting experiments were repeated three times on separate days, independently (n = 3). (TIF) [file pone.0212518.s003.tif]

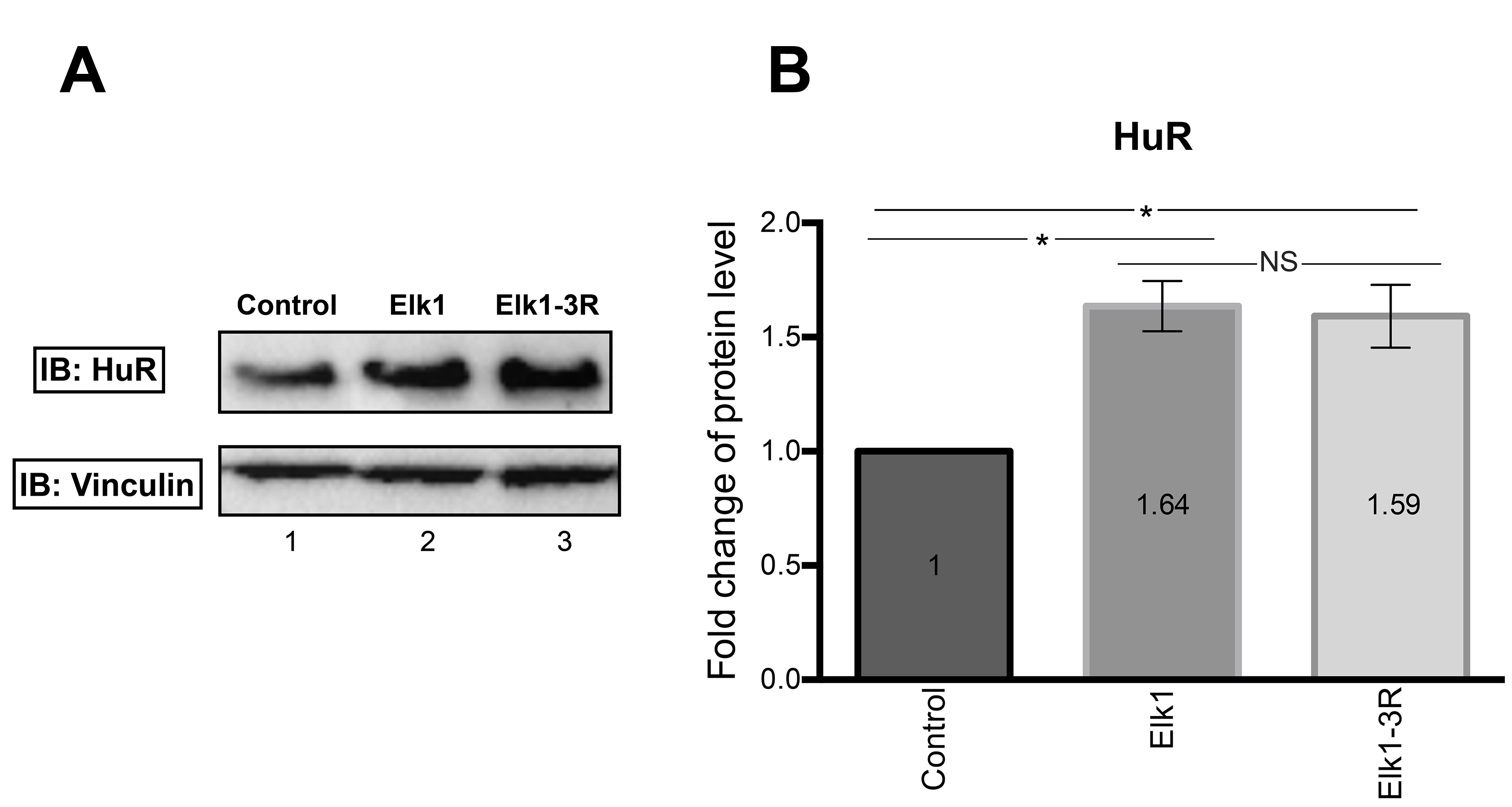

Supplement: S4 Fig — (A) Western blotting results of HuR. Lane 1: untransfected control cell lysate, Lane 2: Elk1 overexpressed cell lysate, Lane 3: Elk1-3R overexpressed cell lysate. In comparison to control cells, both Elk1 and Elk1-3R overexpression result in increased HuR level indicated in upper part of the figure. Lower part shows vinculin expression as loading control. (B) Quantification result of normalized HuR level. HuR level was significantly increased in Elk1 and Elk1-3R overexpressed cell lysates. SEM values are 0, 0.11, and 0.14, respectively. Transfection and Western blotting experiments were repeated three times on separate days, independently (n = 3). (TIF) [file pone.0212518.s004.tif]

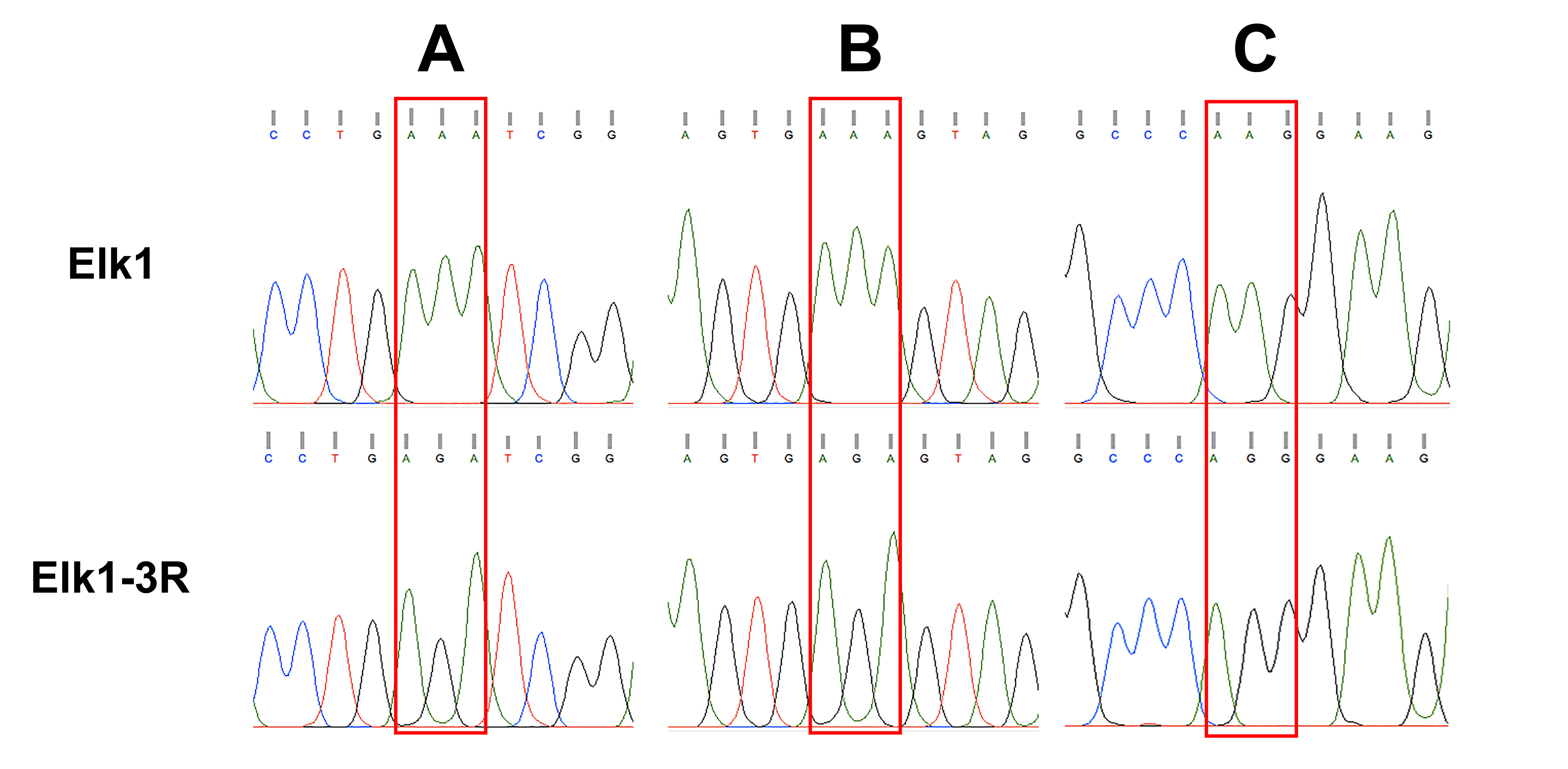

Supplement: S5 Fig — Upper part of the figure represents Elk1 chromatograms, while lower part indicates Elk1-3R. Lysine amino acids located on 230 (A), 249 (B), 254 (C) residues were converted into arginines to generate Elk1-3R construct. (TIF) [file pone.0212518.s005.tif]

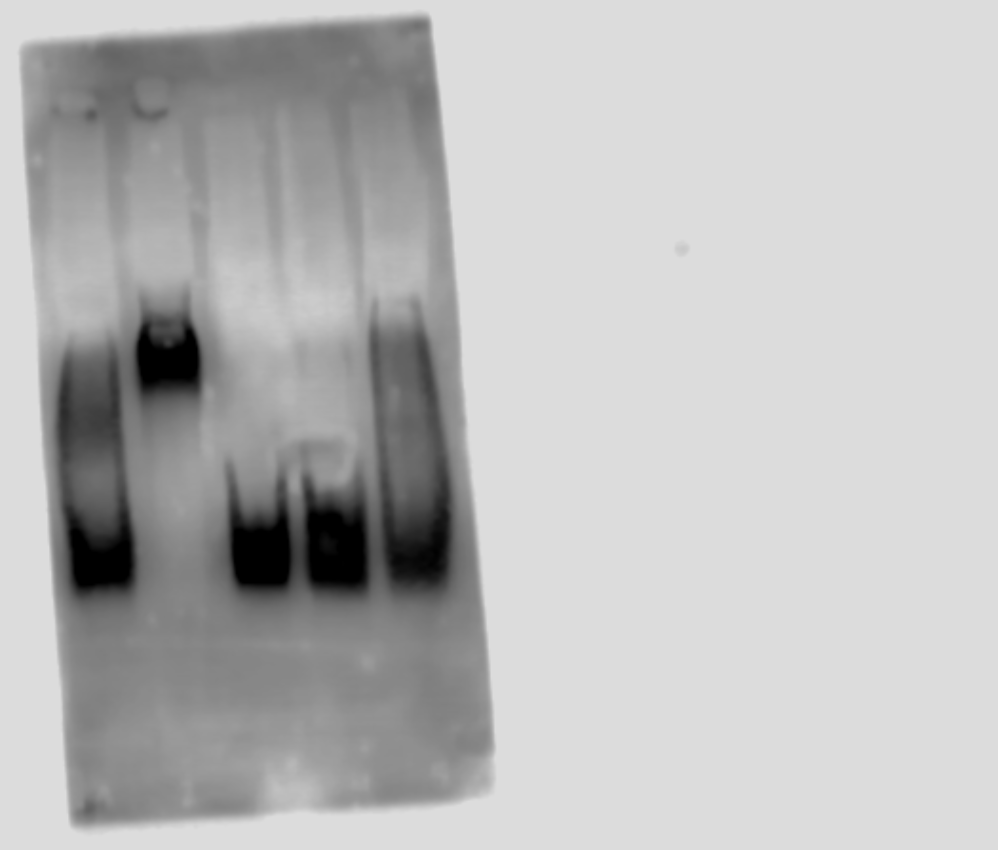

Supplement: S1 Data — (TIF) [file pone.0212518.s006.tif]

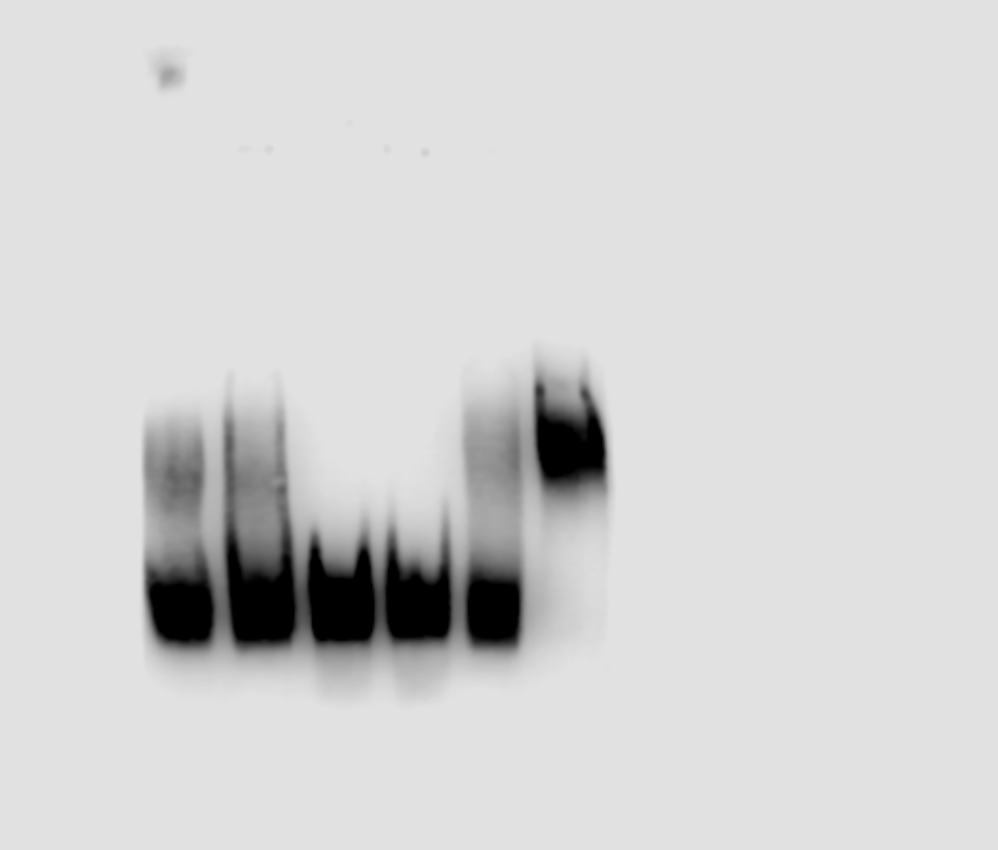

Supplement: S2 Data — (TIF) [file pone.0212518.s007.tif]

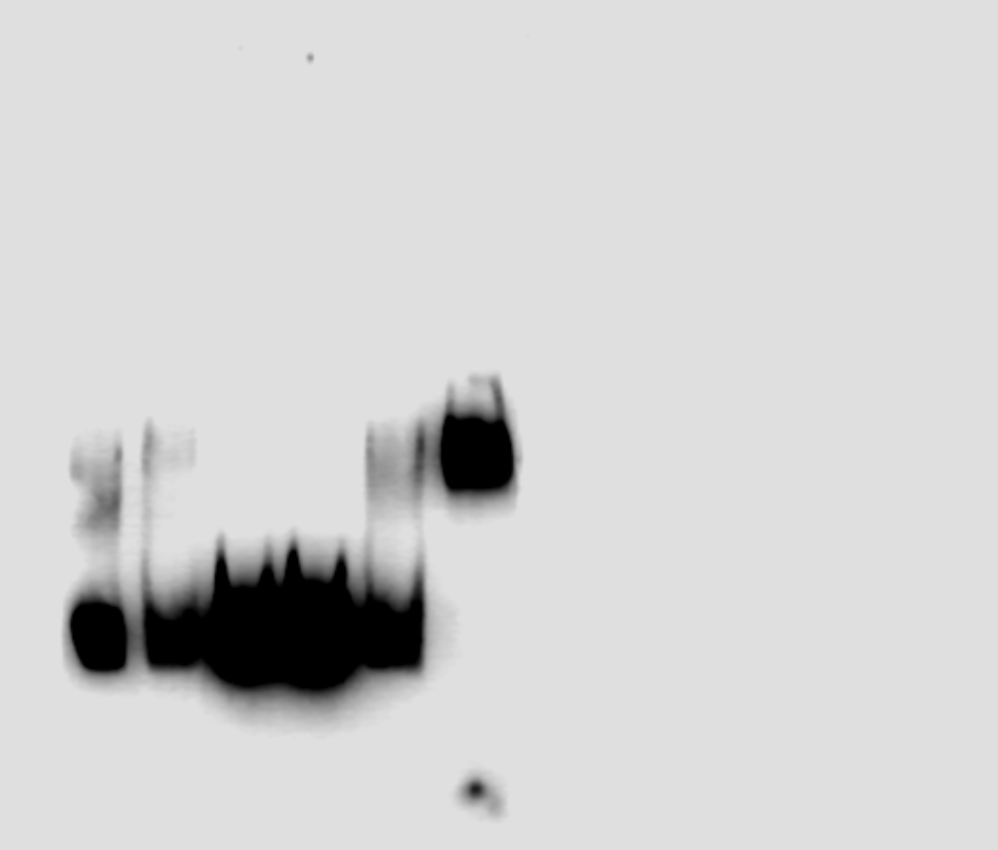

Supplement: S3 Data — (TIF) [file pone.0212518.s008.tif]

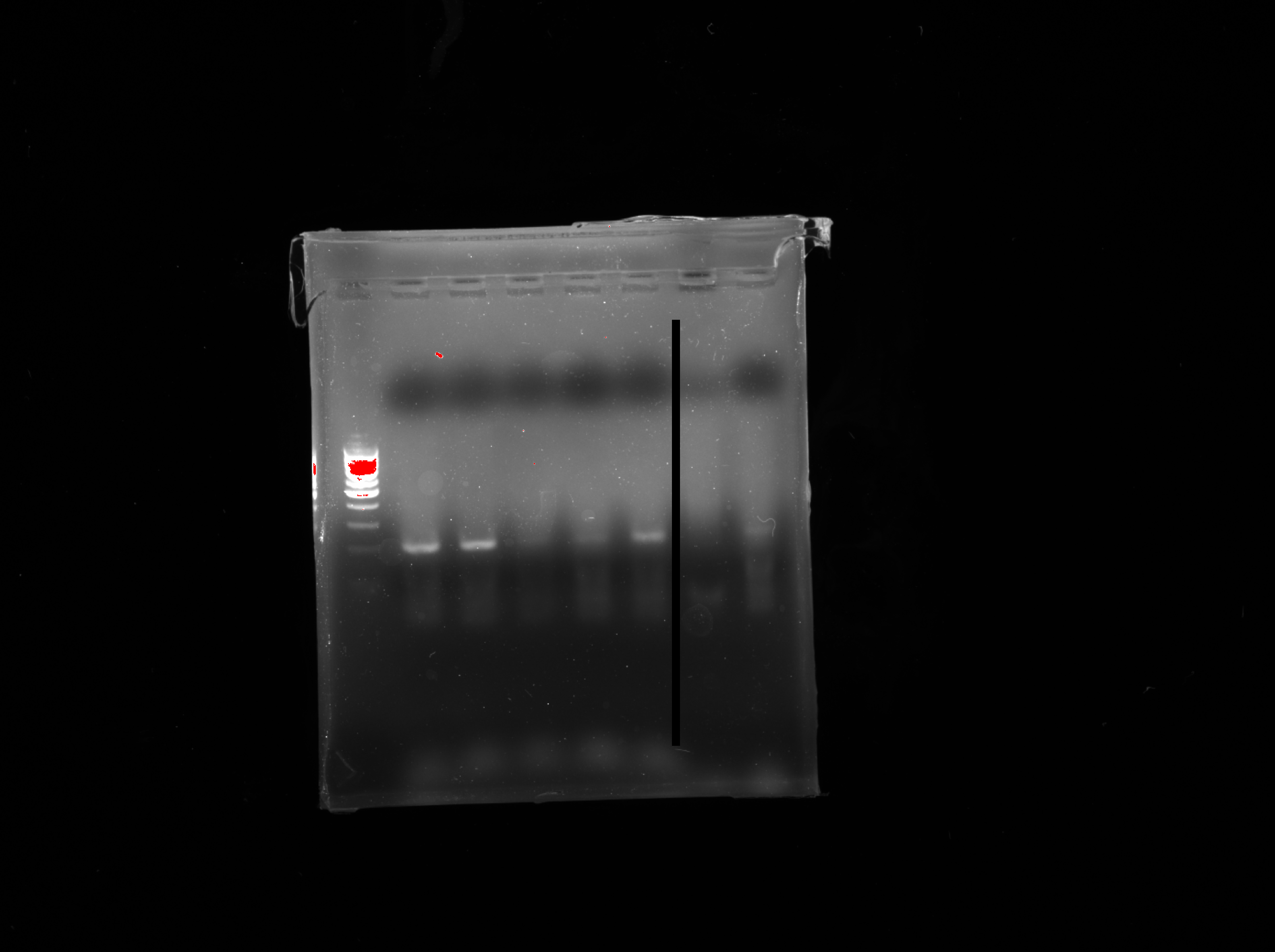

Supplement: S4 Data — Last 2 wells includes irrelevant samples, thus, they were denoted by vertical black line by using Adobe Photoshop CS6 software. (TIF) [file pone.0212518.s009.tif]

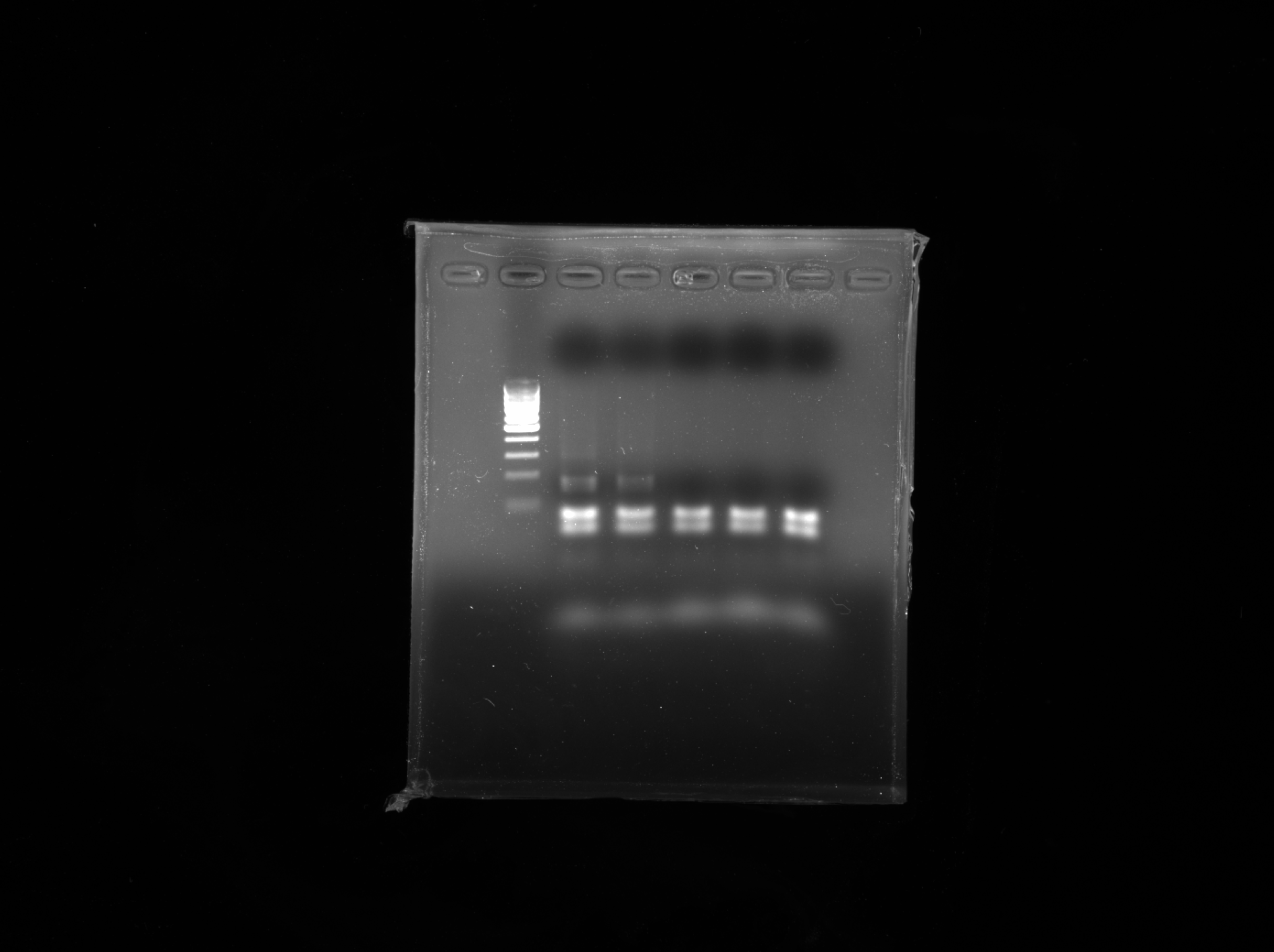

Supplement: S5 Data — (TIF) [file pone.0212518.s010.tif]

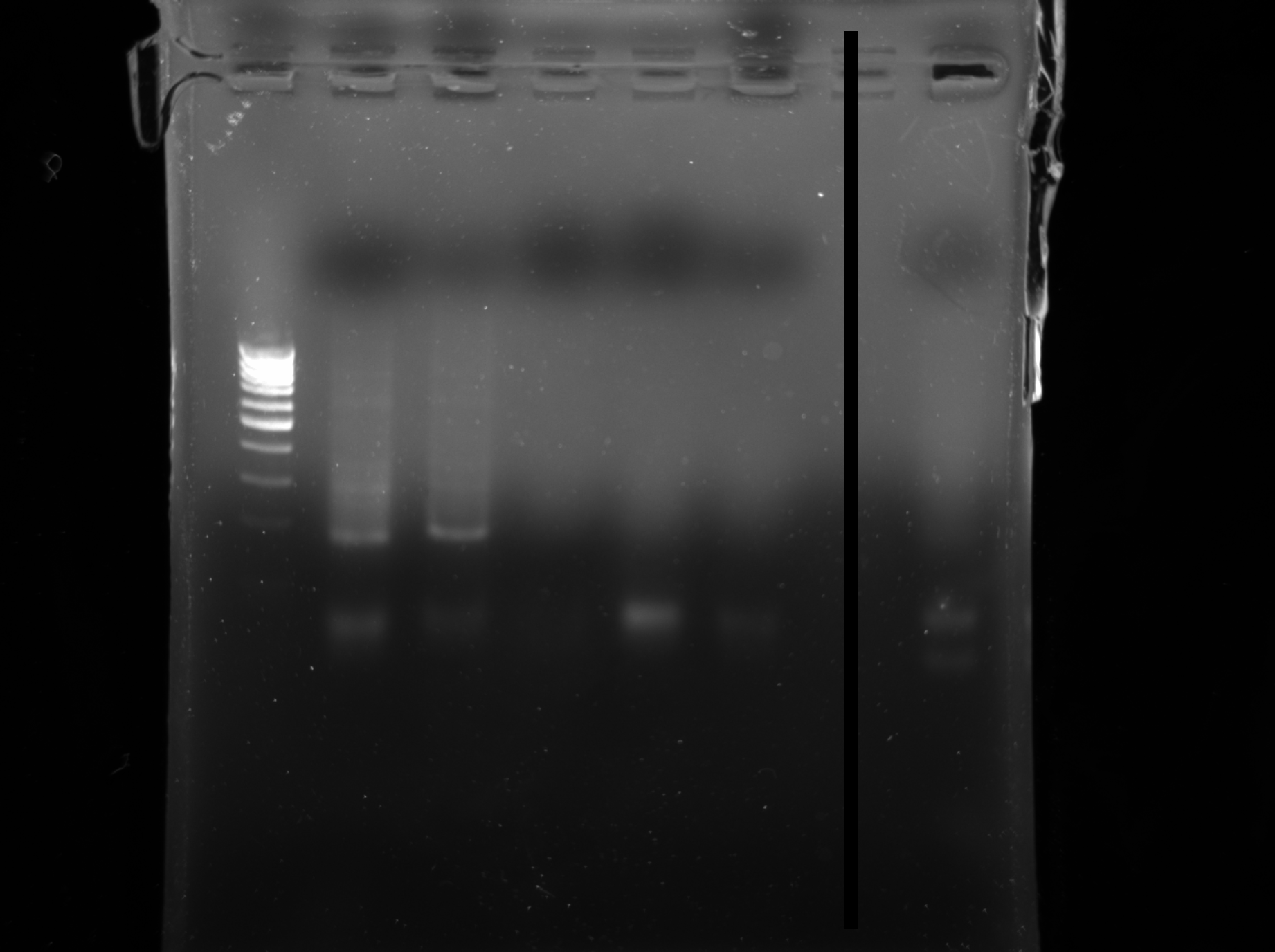

Supplement: S6 Data — Last well includes irrelevant sample, thus, it was denoted by vertical black line by using Adobe Photoshop CS6 software. (TIF) [file pone.0212518.s011.tif]

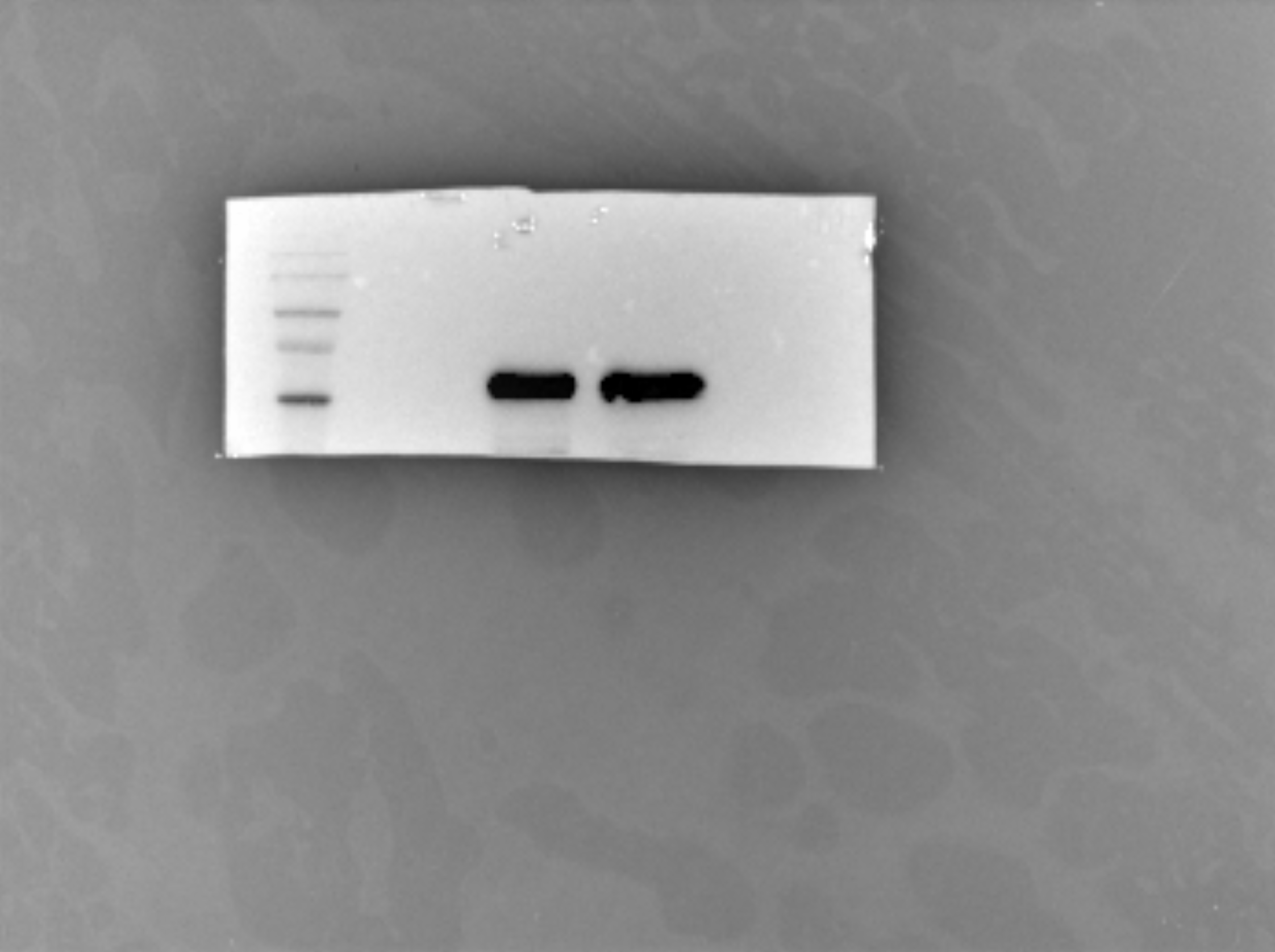

Supplement: S7 Data — Color Prestained Protein Standard, Broad Range (NEB) was used as molecular marker. (TIF) [file pone.0212518.s012.tif]

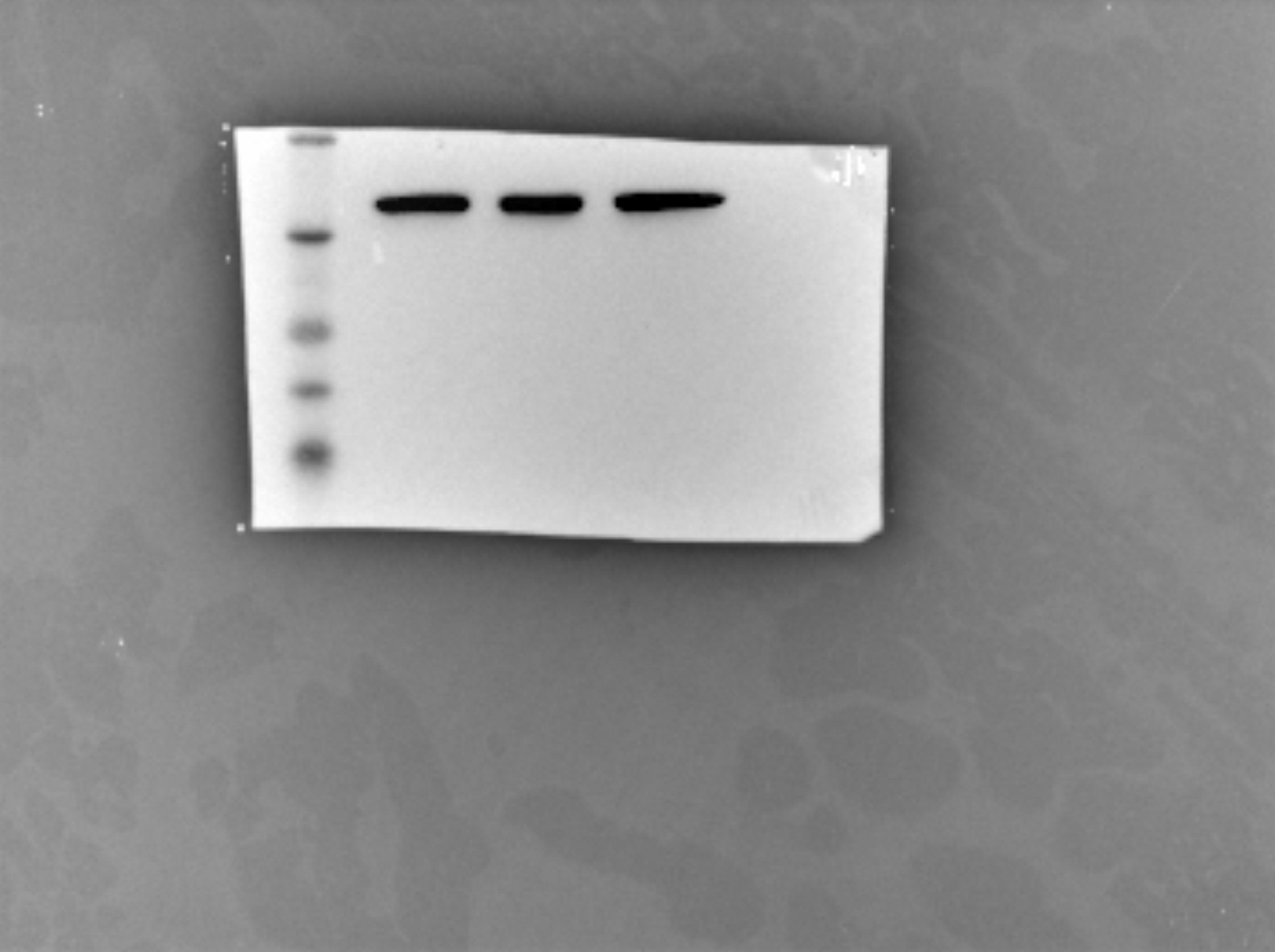

Supplement: S8 Data — Color Prestained Protein Standard, Broad Range (NEB) was used as molecular marker. (TIF) [file pone.0212518.s013.tif]

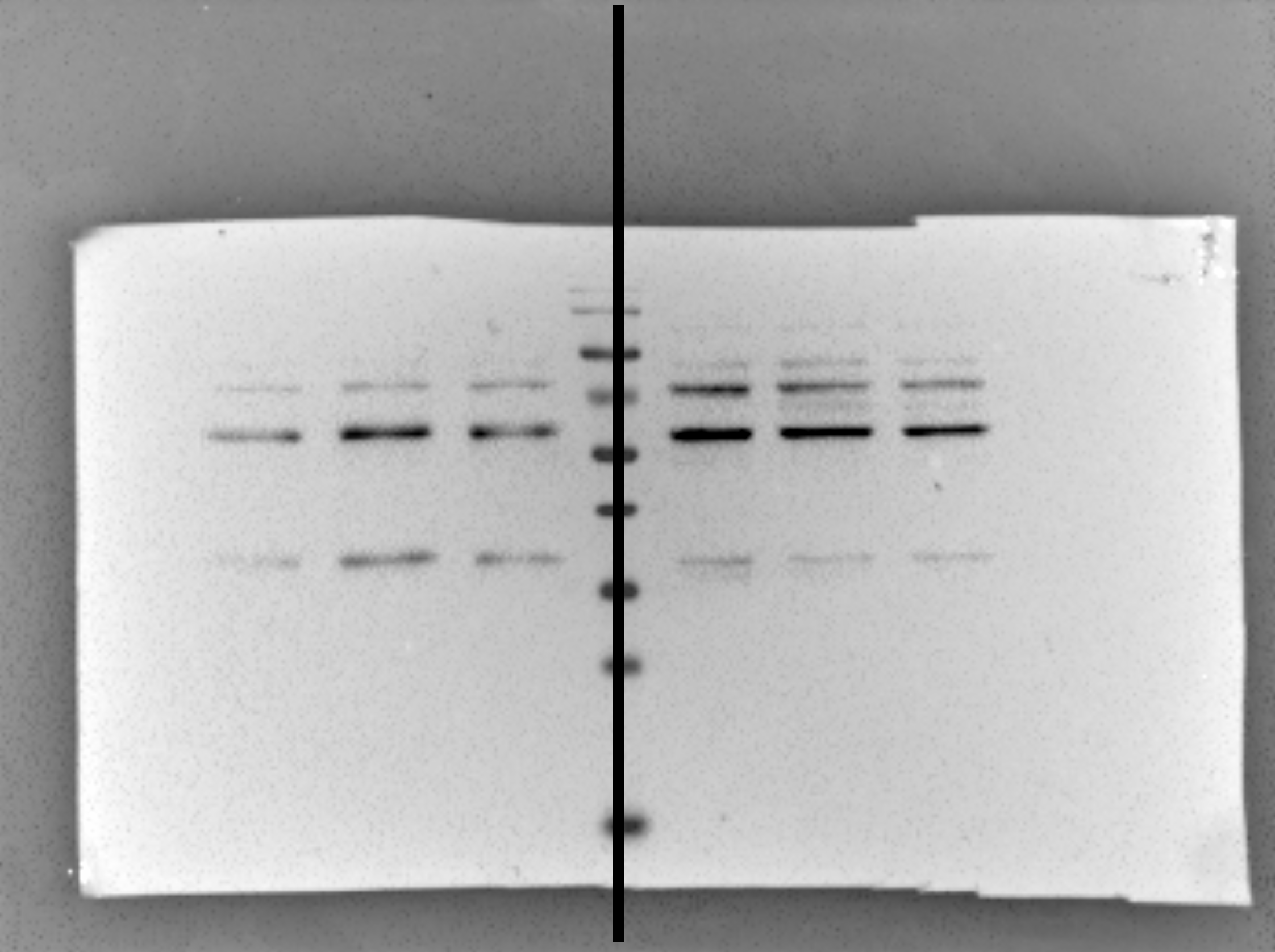

Supplement: S9 Data — The wells on the left side of molecular marker includes irrelevant lysates of another cell type, however, they had been used in order to reveal whether non-specific bands were still present in those samples. Since the antibody was polyclonal, non-specific bands were obtained in each experiment. However, single band in each well was located in between 80 kDa and 58 kDa as near to 58 kDa, also, it had the most strong intensity. Since katanin-p60 is expected as 60 kDa, the band mentioned above was chosen unhesitantly. Color Prestained Protein Standard, Broad Range (NEB) was used as molecular marker. The wells on the left site of molecular marker were denoted by vertical black line by using Adobe Photoshop CS6 software. (TIF) [file pone.0212518.s014.tif]

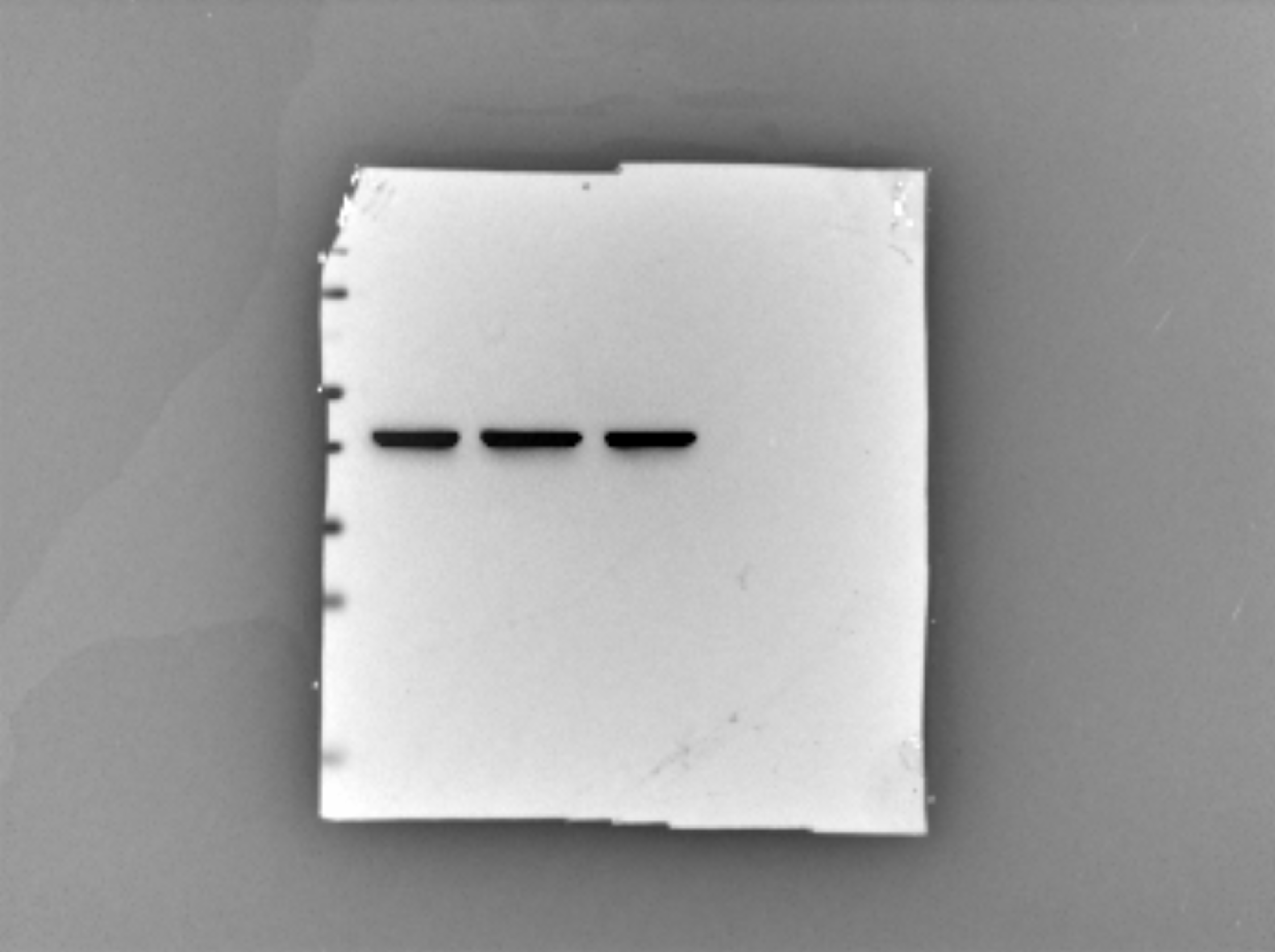

Supplement: S10 Data — (TIF) [file pone.0212518.s015.tif]

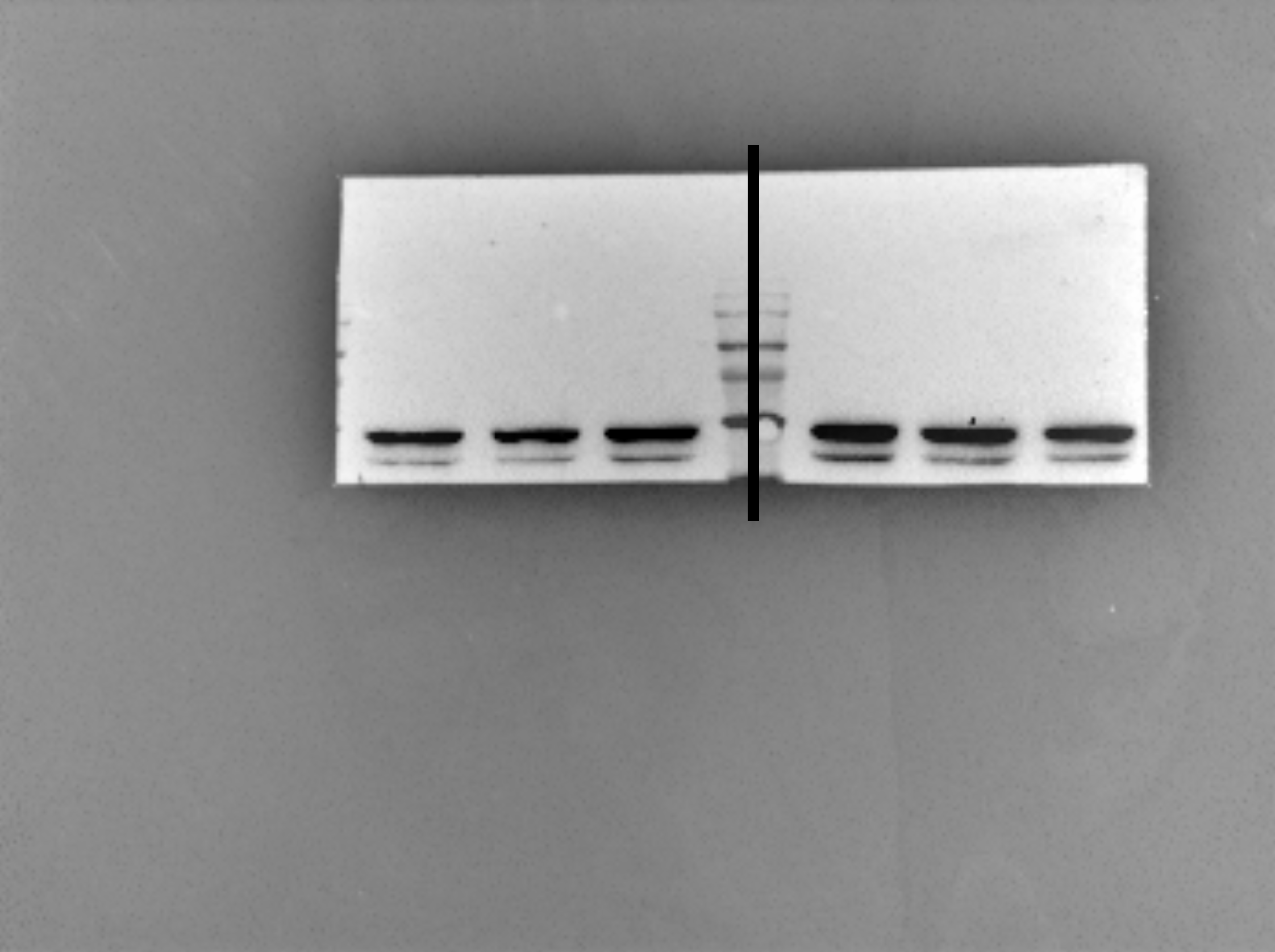

Supplement: S11 Data — Even though the wells on the left side of molecular marker included same samples, they were denoted by vertical line by using Adobe Photoshop CS6 software, as they were not represented in main figure. The bands which were present under our specific bands could be another isoform of spastin instead of non-specific bands, since their level were also decreased. Color Prestained Protein Standard, Broad Range (NEB) was used as molecular marker. (TIF) [file pone.0212518.s016.tif]

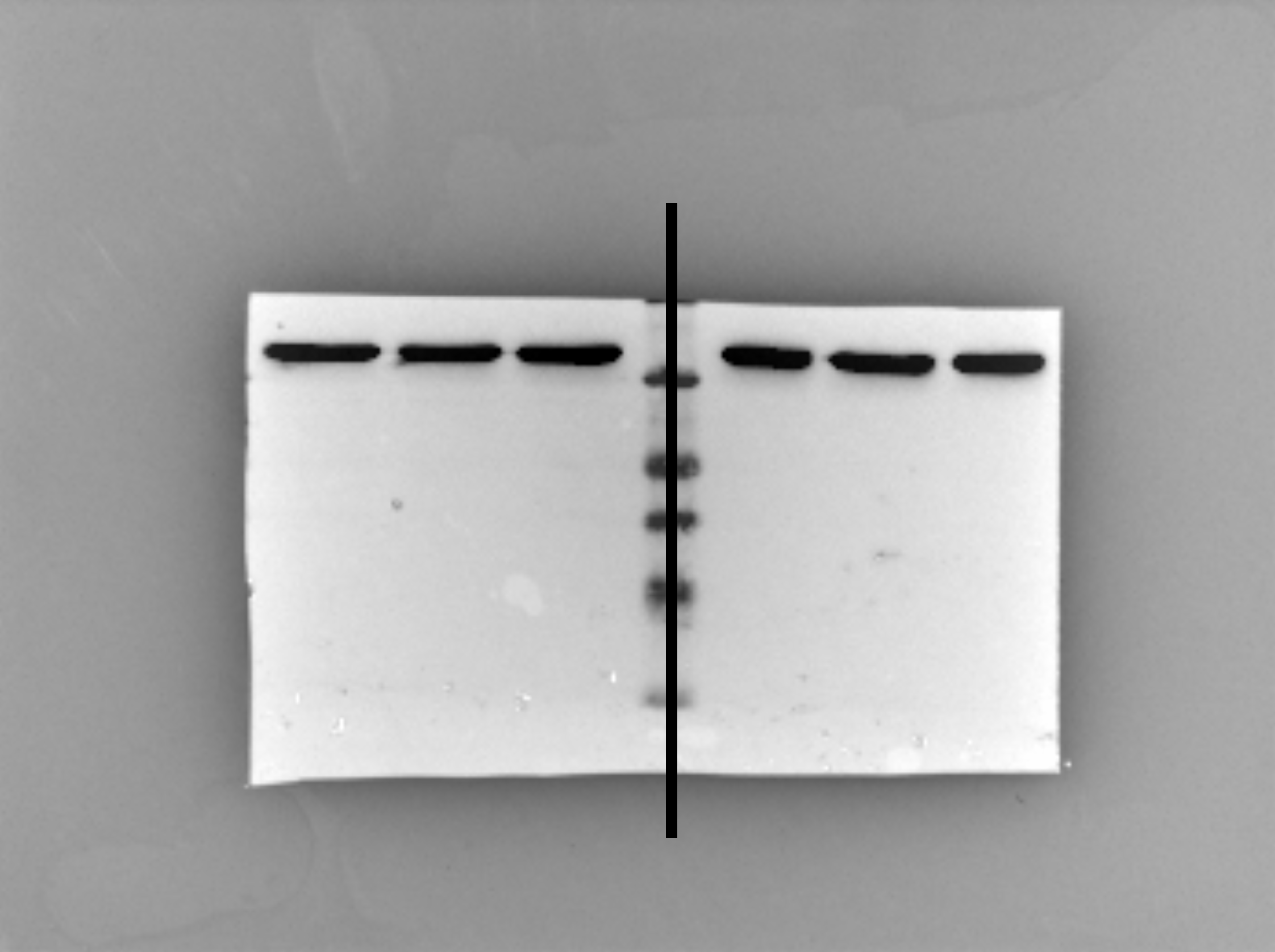

Supplement: S12 Data — Even though the wells on the left side of molecular marker included same samples, they were denoted by vertical line by using Adobe Photoshop CS6 software, as they were not represented in the main figure. Color Prestained Protein Standard, Broad Range (NEB) was used as molecular marker. (TIF) [file pone.0212518.s017.tif]

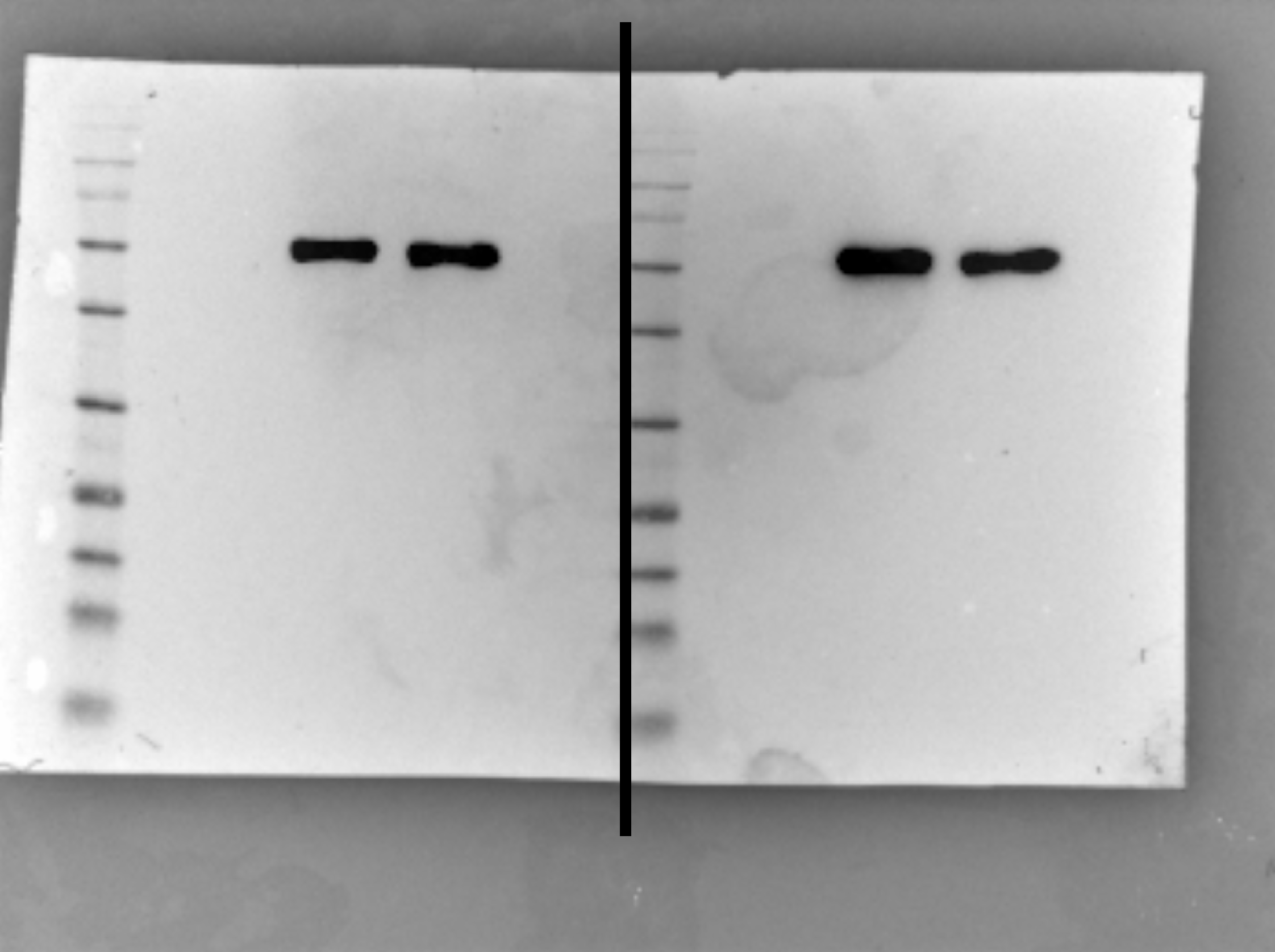

Supplement: S13 Data — The wells on the right side were denoted by vertical line by using Adobe Photoshop CS6, since they were irrelevant to the our experiment. Color Prestained Protein Standard, Broad Range (NEB) was used as molecular marker. (TIF) [file pone.0212518.s018.tif]

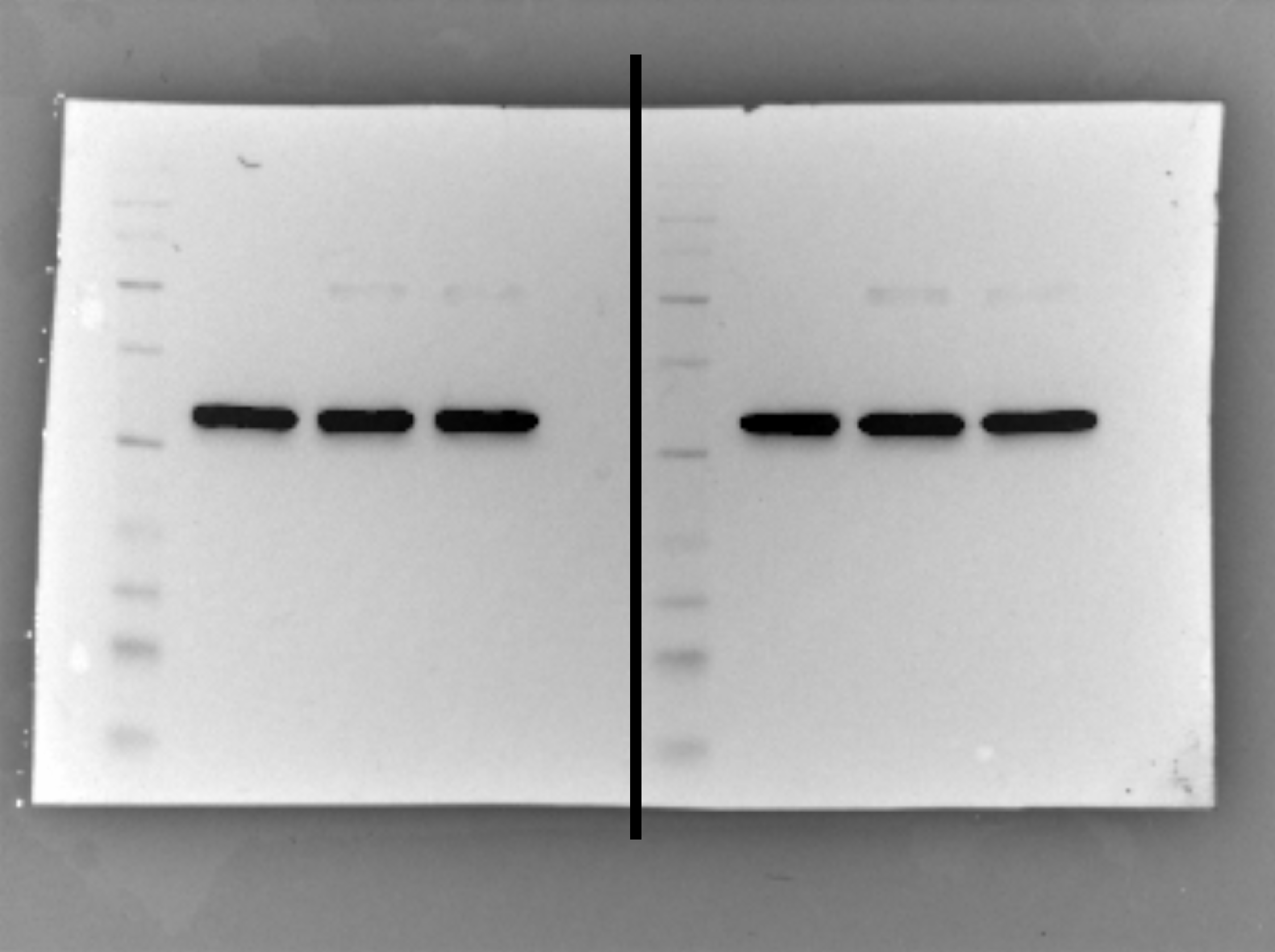

Supplement: S14 Data — The wells on the right side were denoted by vertical line by using Adobe Photoshop CS6, since they were irrelevant to our experiment. Color Prestained Protein Standard, Broad Range (NEB) was used as molecular marker. (TIF) [file pone.0212518.s019.tif]

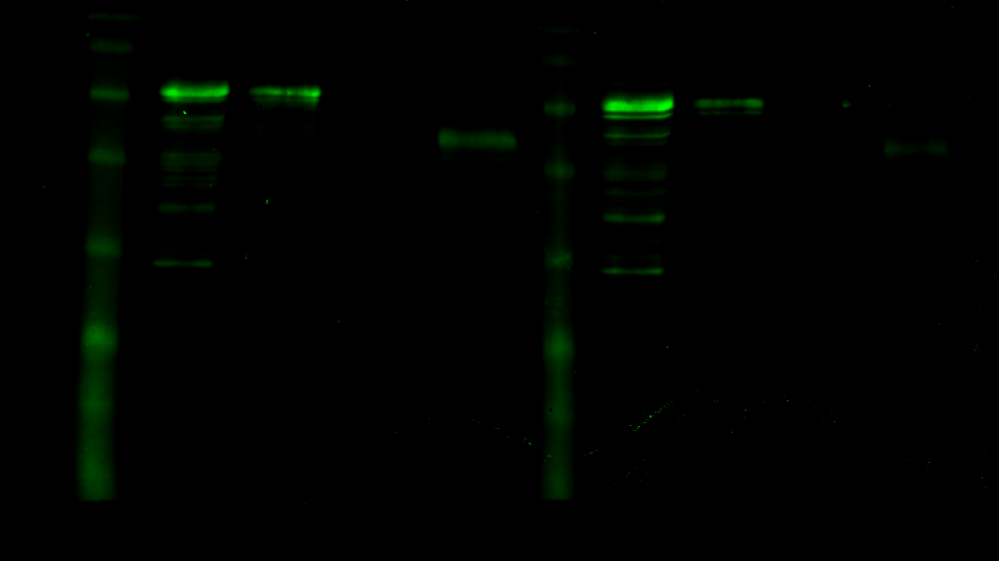

Supplement: S15 Data — Color Prestained Protein Standard, Broad Range (NEB) was used as molecular marker. (TIF) [file pone.0212518.s020.tif]

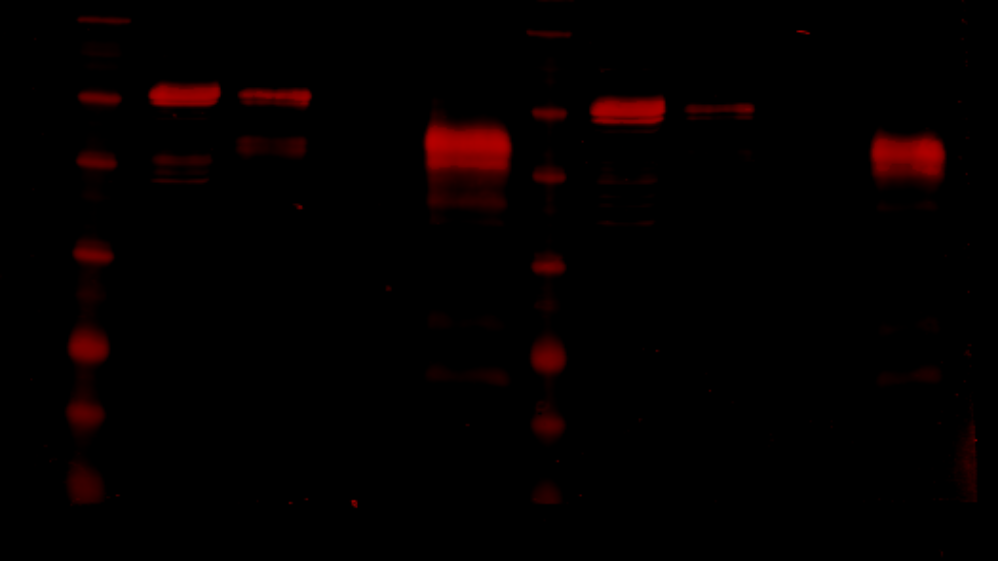

Supplement: S16 Data — Color Prestained Protein Standard, Broad Range (NEB) was used as molecular marker. (TIF) [file pone.0212518.s021.tif]

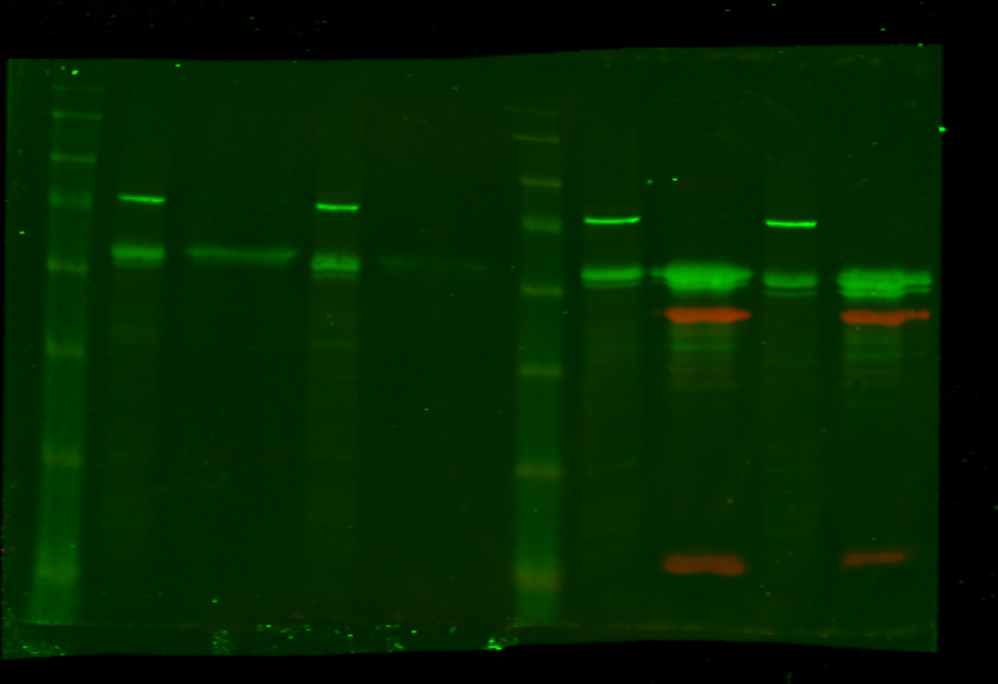

Supplement: S17 Data — Color Prestained Protein Standard, Broad Range (NEB) was used as molecular marker. (TIF) [file pone.0212518.s022.tif]

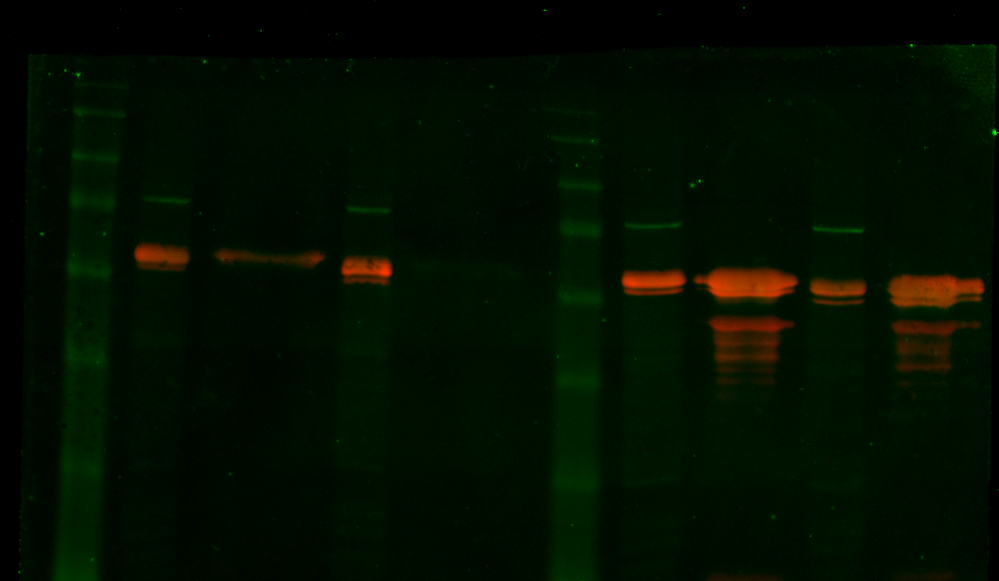

Supplement: S18 Data — Color Prestained Protein Standard, Broad Range (NEB) was used as molecular marker. (TIF) [file pone.0212518.s023.tif]

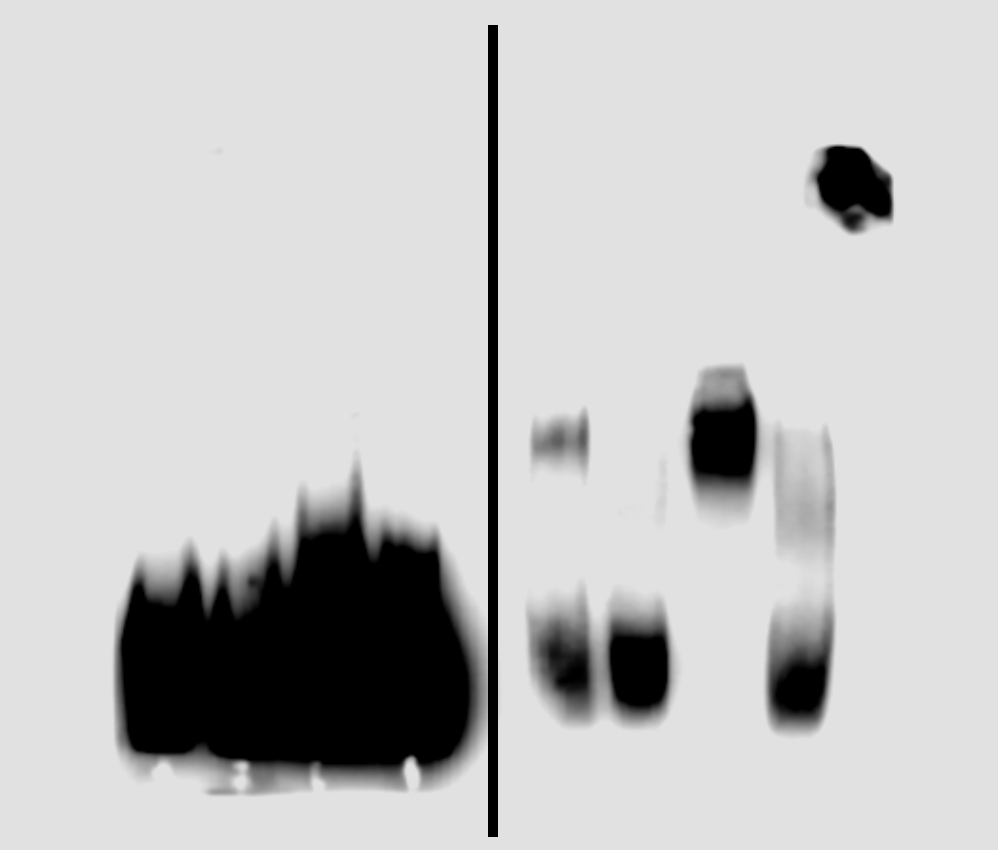

Supplement: S19 Data — The first 4 wells were denoted by vertical line by using Adobe Photoshop CS6, since they included irrelevant samples. (TIF) [file pone.0212518.s024.tif]

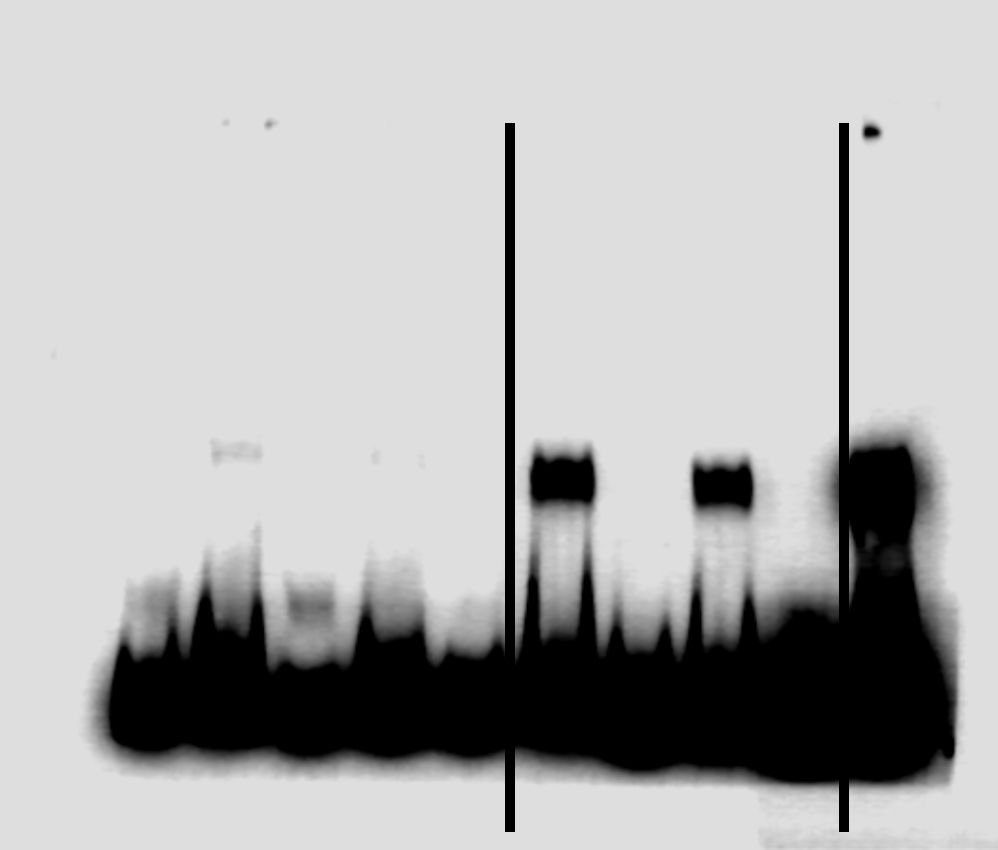

Supplement: S20 Data — Since first 5 wells included irrelevant samples and the last well included higher probe concentration than other wells, they were denoted by vertical lines by using Adobe Photoshop CS6. The main figure in manuscript has been represented in between the lines. (TIF) [file pone.0212518.s025.tif]
